# Microplastics as Emerging Contaminants and Human Health: Exploring Functional Nutrition in Gastric–Colon–Brain Axis Cancer

**DOI:** 10.3390/toxics13060438

**Published:** 2025-05-26

**Authors:** Maria Scuto, Cinzia Maria Grazia Lombardo, Bruna Lo Sasso, Eleonora Di Fatta, Raffaele Ferri, Angela Trovato Salinaro

**Affiliations:** 1 Department of Medicine and Surgery, “Kore” University of Enna (UKE), 94100 Enna, Italy; mariaconcetta.scuto@unikore.it (M.S.); bruna.losasso@unikore.it (B.L.S.); 2 Department of Biomedical and Biotechnological Sciences, University of Catania, 95123 Catania, Italy; cinzia.lombardo@unict.it; 3 OASI Research Institute-IRCCS, 94018 Troina, Italy; edifatta@oasi.en.it (E.D.F.); rferri@oasi.en.it (R.F.)

**Keywords:** microplastics, emerging contaminants, cancer, functional nutrients, flavonoids, oxidative stress, brain toxicity, artificial intelligence, gastric–colon–brain axis

## Abstract

Microplastics (MPs), emerging contaminants of significant global concern, have a substantially increased environmental impact due to their biological persistence and accumulation in the body. Exposure to MPs has been associated with oxidative stress, systemic inflammation, and cellular dysfunction, notably affecting critical tissues such as the stomach, colon, and brain. This review explores the correlation between MPs and cancer risk along the gastric–colon–brain axis, identifying the signaling pathways altered by MP exposure. Furthermore, it highlights the role of functional nutrition and bioactive flavonoids—including chlorogenic acid, coumaric acid, and naringin—as well as the use of highly bioavailable combined polyphenol nanoparticles as potential detoxifying agents. Functional nutrients are effective in enhancing cellular resilience against reactive oxygen species (ROS) production and MP-induced toxicity, offering protective effects at the gastric, intestinal, and brain barriers. Activation of the Nrf2 pathway by bioactive compounds promotes the expression of detoxifying enzymes, suggesting a promising nutritional strategy to mitigate MP-related damage. This review underscores how functional nutrition may represent a viable therapeutic approach to reduce the harmful effects of MP exposure. The integration of advanced technologies—such as microfluidic systems, organ-on-a-chip platforms, and machine learning—and the identification of key molecular targets lay the foundation for developing preventive and personalized medicine strategies aimed at lowering the risk of environmentally induced carcinogenesis.

## 1. Introduction

**Figure 1:**
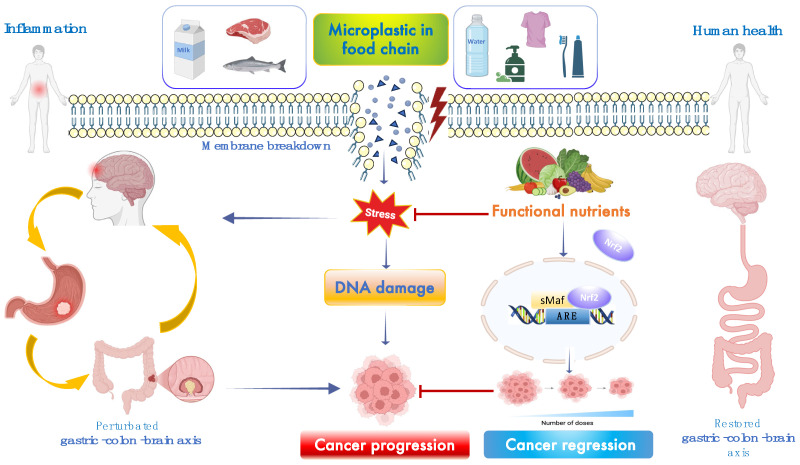
**Potential mechanisms of MP-induced cancer progression.** Exposure to MPs induces various biological responses, including DNA damage, mitochondrial dysfunction, inflammation, and apoptosis. Oxidative stress and pro-inflammatory signaling pathways activated by MP exposure may promote tumorigenesis [7,13,15]. Functional food nutrients may counteract these effects and support cancer regression along the gastric–colon–brain axis [16,25,40]. Created in BioRender 2025. Scuto, M. (2025); https://BioRender.com/sb7m27f (accessed on 12 May 2025).

**Figure 2:**
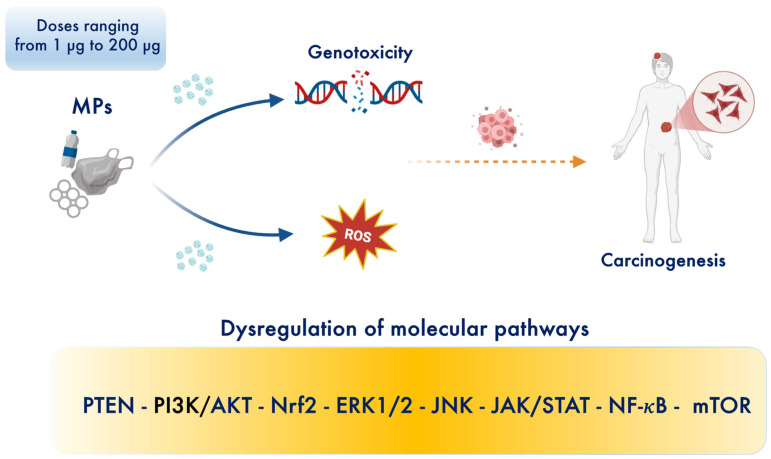
MPs induce dose-dependent systemic genotoxicity by disrupting multiple cellular and molecular pathways [42,43]. Created in BioRender. Scuto, M. (2025); https://BioRender.com/btg0i9q (accessed on 12 May 2025).

The increasing prevalence of emerging contaminants, particularly microplastics (MPs), poses a significant risk to human health. MPs are now ubiquitous in ecosystems, contaminating various foods and drinking water. The escalating production of plastics—exceeding one million tons annually—is a global concern due to the persistence and bioaccumulation of MPs, defined as plastic particles ≤ 5 mm. These particles, composed of polymers such as polyethylene (PE), polypropylene (PP), poly (ethylene terephthalate) (PET), and polystyrene (PS), vary in size, shape, and color [1]. Human exposure to MPs occurs via gastrointestinal intake, dermal contact, and pulmonary inhalation, with oral ingestion being the most common route—resulting in the intake of tens of millions of MPs annually (several milligrams per day). Ingested MPs can induce acute and chronic gastric inflammation, disrupt the gut barrier, and cause dysbiosis (Figure 1). For example, exposure to polymethyl methacrylate MPs has been shown to trigger senescence in gastric mucosal epithelial cells through reactive oxygen species (ROS) overproduction, pro-inflammatory mediators, and impaired DNA repair by inhibiting the NHEJ pathway in a concentration-dependent manner [2]. Furthermore, MPs can cause neuroinflammation, cognitive impairment, and neurotoxic effects in the brain, potentially contributing to neurodegeneration and brain tumors [3]. The brain–gut axis plays a crucial role in regulating diet and hygiene practices through communication between the nervous and endocrine systems. Oxidative stress, caused by an imbalance between ROS production and cellular detoxification or repair mechanisms, leads to the production of peroxides and free radicals that damage cellular components, including proteins, lipids, DNA, and RNA [4,5] (Figure 1). While ROS have physiological roles, excessive production of free oxygen radicals can damage tissues. Dietary MP exposure in adult zebrafish inhibited antioxidant pathways, decreased glutathione reductase (GR) activity, and led to potential ROS accumulation in offspring [6]. In fetal brains, excessive ROS induced by MPs can cross the blood–fetal barrier, depleting antioxidant capacity, inducing neural cell apoptosis, and reducing GABA synthesis [7]. MPs and other pollutants disrupt cellular pro-oxidant/antioxidant homeostasis, inducing oxidative stress, chronic inflammation, altered gene expression, and cell proliferation in a size-, dose-, and time-dependent manner [8]. Consequently, MPs play a pivotal role in tumor initiation, promotion, and progression in vitro and in vivo [9,10]. Cancer remains a leading cause of death globally, with approximately 19.3 million new cases and 10 million deaths in 2020 [11]. Bibliometric analyses have shown a strong correlation between cancer development and exposure to environmental contaminants and toxins [12]. According to recent findings, MP-induced oxidative stress affects signaling pathways involved in cell proliferation and migration [13], including the epidermal growth factor receptor (EGFR) pathway and key signaling protein pathways such as Nrf2 (Figure 1), RAS/RAF, ERK1/2, MEK, phospholipase C, protein kinase C, PI3K/Akt, JAK/STAT, and TGF-β pathways [14]. MPs also alter the expression of the p53 tumor suppressor gene, exacerbating carcinogenesis [15]. Oxidative stress, DNA damage, and apoptosis deregulation are among the biochemical mechanisms proposed to link chemical contaminant exposure with adverse health effects such as immunosuppression, neurological diseases, and cancer (Figure 1). Despite advances in understanding neoplastic transformation and diagnostics, studies evaluating the potential impact of MPs on cancer development—and the preventive or therapeutic effects of functional nutrients in mitigating oxidative damage along the gastric–colon–brain axis—remain limited. Therefore, effective alternative therapeutic strategies are urgently needed to combat the cancer risk potentially induced by MP bioaccumulation in these vital organs. Recognizing the intricate relationship between food and health, functional nutrition plays a central role in preventing chronic diseases and maintaining optimal health [16]. Identifying functional nutrients as food supplements to protect barrier integrity and preserve gastric, colon, and brain health against MP-induced stress and damage is a major challenge for modern civilization. Functional nutrition, particularly the use of active flavonoids, is gaining attention for its potential to enhance stress resilience signaling and prevent or reverse chronic inflammatory disorders associated with oxidative stress (Figure 1) [17,18,19,20,21,22,23,24,25,26,27,28]. Today, functional nutrition can be considered a therapeutic response to MP-induced damage. Several lines of evidence suggest that flavonoids and other active nutrients reinforce intestinal and neuronal barriers and activate antioxidant pathways. Emerging studies report that functional nutrients activate the Nrf2 pathway and phase II detoxifying proteins such as heme oxygenase-1 (HO-1), heat shock protein 70 (Hsp70), and sirtuin-1 (Sirt1), thioredoxin (Trx), as well as enzymes including superoxide dismutase (SOD), catalase (CAT), NADPH:quinone oxidoreductase (NQO1), glutathione S-transferase (GST), glutathione peroxidase (GPx), glutathione reductase (GR), and forkhead box class O (FoXO) [29,30,31,32,33,34,35,36,37,38]. These molecules mitigate excessive MP-induced stress and cellular damage by converting electrophilic molecules and reactive free radicals into nontoxic substances that can be easily excreted. This detoxification process inhibits oxidative damage and attenuates carcinogen-derived reactive metabolites triggered by MP bioaccumulation, which can lead to gene mutations, instability, and carcinogenesis along the gastric–colon–brain axis. Detoxifying enzymes are regulated by the antioxidant response element (ARE) in the 5′ upstream promoter region of these genes. The Nrf2 pathway—a cytoplasmic stress resilience system—is central to this transcriptional response and a primary molecular target for chemoprotective agents [39]. Electrophilic compounds or functional flavonoids, including chlorogenic acid, coumaric acid, naringin, naringenin, nobiletin, luteolin, and highly bioavailable polyphenol–nanoparticle complexes, can activate the cytoprotective Nrf2 pathway. This pathway plays a key role in chemoprevention, particularly by blocking the initiation stage of cancer proliferation [40,41]. However, constitutive activation of the Nrf2 pathway due to MP-induced mutations can be a double-edged sword, as its downstream resilience genes may also contribute to cancer cell growth by promoting anti-senescence, proliferation, anti-apoptosis, autophagy deficiency, and resistance to chemotherapy or radiotherapy [42]. Indeed, it has been reported that MPs induce stress and systemic genotoxicity (Figure 2) by altering multiple cellular and molecular pathways, including the MAPK signaling pathway (RTK, RAS, ERK, JNK, P38, NRF2, TNF-α) and the PI3K–AKT pathway (PI3K, AKT, MDM2, P53, BAD), both associated with carcinogenesis [42].

Functional flavonoids may promote apoptotic cell death in preneoplastic or neoplastic cells through various growth-inhibitory mechanisms, including cytochrome c and caspase activation, cell cycle arrest, and modulation of signaling pathways that inhibit MP-mediated tumor progression. This review aims to explore functional drug–food candidates that activate detoxification processes catalyzed by stress resilience genes and proteins to attenuate oxidative stress, toxicity, and carcinogenic pathways triggered by emerging contaminants such as MPs. It also examines the potential development of cancer risk along the gastric–colon–brain axis. Furthermore, it discusses innovative platforms for detecting cellular MPs and predicting their toxicity, with the goal of discovering promising preventive, precision, and personalized nutritional therapeutic strategies for human health. By focusing on the gastric–colon–brain axis—three key systems of the human organism—this review highlights how damage caused by MPs can propagate systemically. This is a critical and underexplored area. The development of new cellular detection strategies for MPs and the identification of biomarkers of exposure and damage could pave the way for new therapeutic approaches in the context of personalized medicine.

## 2. Microplastics as Emerging Contaminants for Cancer Risk in Gastric–Colon–Brain Axis

**Figure 3:**
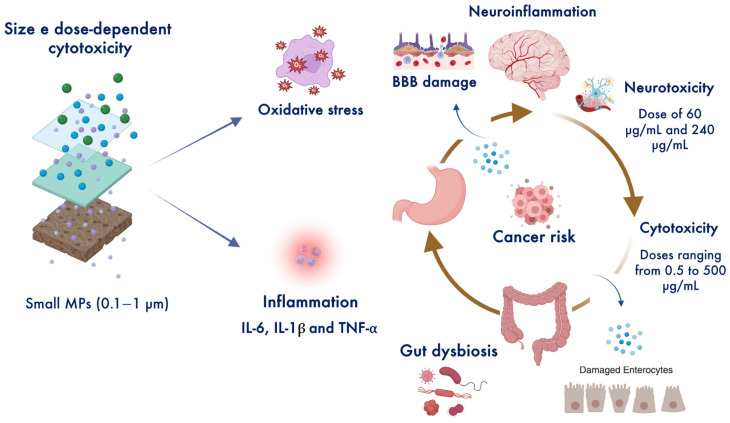
Intracellular accumulation of MPs exposes cells to carcinogenic agents, inducing dose-dependent cytotoxicity and neurotoxicity [43,44,45,46]. Created in BioRender. Scuto, M. (2025); https://BioRender.com/8pr9w55 (accessed on 12 May 2025).

Due to the increasing global prevalence of plastic pollution, the human body is exposed daily to significant quantities of MPs. Continuous oral ingestion and the subsequent accumulation of MPs in vital tissues and organs raise concerns about potential short- and long-term health effects, including the progression of chronic inflammatory diseases into cancer [43] (Figure 3). Understanding the potential impact of MPs and the underlying mechanisms of MP-induced carcinogenesis along the gastric–colon–brain axis is therefore of significant interest [44]. Table 1 illustrates the correlations between signaling pathways and cancer risk in in vitro and animal models based on MP size and dose. MPs that induce oxidative damage and inflammation may exacerbate genetic mutations and contribute to cancer development [12,13,14,15,42]. Currently, no clinical studies have directly examined the relationship between MPs and cancer within the gastric–colon–brain axis. Thus, the actual dose of MPs that may lead to carcinogenesis in humans remains to be validated and confirmed. Our hypothesis is that chronic exposure and/or daily intake of small amounts of MPs—particularly at nano- and micrometer scales and in high doses—may lead to oxidative stress, chronic inflammation, and, ultimately, an increased long-term cancer risk.

**Table 1:** Potential signaling pathways ↑ activated or ↓ inhibited by MPs in experimental models.

Models/Biological Matrix	Pathways	MPs Sizes and Doses	Outcomes	Ref.
Human renal tubular epithelial cells and human testis Cancer cells	↑ TNF-α, TNF-α-R, ↑ RTK, RAS, JNK, ↑ ERK, P38, ↑ PI3K-AKT ↑ NRF2 ↑ MAPK	50 nm 200 μg/mL for 24 h	PS-NPs cross into cells through endocytotic vesicles and affect cellular micro-structures and pathways related to cancer progression and metastasis.	[42]
Human gastric cancer cells	↑ ASGR2 ↑ CD44 ↑ N-cadherin ↑ PD-L1	10 μm PS-MPs, 8.61 × 10^5^ particles/mL	PS-MPs exposure induce invasion, migration and multidrug resistance.	[43,44,45,46,47]
Mice		1.72 × 10^4^ particles/mL orally administered for 4 weeks	PS-MPs accumulated in gastric tissue induced resistance to chemo- and monoclonal antibody-therapy.	
Gastric cancer cells	Not specified	60 nm PS-NPs and 500 nm PS-MPs at a dose of 200, 400, 600 mg/L	Induce intracellular ROS and genotoxicity.	[48]
Drinking water	Not specified	0.125–0.15 mm, dose of 10 mg	MP-sorbed PHE and their derivatives increase gastrointestinal toxicity and human cancer risk particularly to higher levels of 10^−4^ in both adults and children.	[49]
Human colorectal cancer cells and spheroid cells	Not specified	0.25 and 1 μm and dose of 0.1, 1, and 10 μg mL	PS-MPs smaller than 1 μm enhance cell migration, potentially promoting metastasis.	[50,51]
Resistant HCT-116 and colorectal SW480 cancer	↑ mTOR/ULK1	60 to 80 nm dose 25 µg/mL	Enhance drug resistance and CRC cancer progression by promoting mTOR-mediated protective autophagy.	[52]
Human colorectal adenocarcinoma caco-2 and HT-29 cells	↑ ROS	0.45 μm dose 0.25–1.0 mg/ml for 48 h	Decrease cell viability and increase oxidative stress, particularly mitochondrial superoxide production dose-dependently.	[53]
Mice	↑ VLA4-VCAM1 ↑ IL-10 ↑ TNF-α ↑ IFN-γ	0.1, 5, and 50 μm 10 mg/L	Long-term oral ingestion of the smallest MPs (0.1 μm) promotes gut epithelium damage and colitis related to depressive-like behaviors.	[54,55,56,57]
Mouse brain neuroblastoma NEURO-2A cells and human choriocarcinoma HLA-G-positive cells	↓ GABA ↓ Claudin 3 ↑ ROS	Size PS-MPs 1000 nm, PS-NPs 100 nm, PS-NP-COOH 100 nm dose 60 μg/mL and 240 μg/mL for 48 h	Promote neurotoxic effects by inducing oxidative stress and apoptosis with GABA depletion.	[7]
Mice	↓ GABA ↑ ROS	Size 100 nm, 1 mg/day via intragastric gavage for 17 consecutive days	Maternal administration of PS-MPs during gestation cross maternal blood-placental barrier and lead to anxiety-like behavior of the progenies and GABA reduction in the prefrontal cortex and amygdala after 8 weeks.	
Human SH-SY5Y neuroblastoma cells	↑ AMPK/ULK1	Size 50 nm, 0.5–500 μg/mL for 28 days	Induce neurotoxicity and mitochondrial dysfunction causing dopaminergic neuron death in a dose-dependent manner.	[58,59,60]
Mice	↑ AMPK/ULK1	250 mg/kg/day by oral gavage		
Human U87 glioblastoma cells	Not specified	Size range of 37–75 μm 0.005 g for 26 days	Long-term exposure to MPs increases the proliferative and migratory capacities with a tendency to aggregate into a cluster of cells (spheroids).	[61]

### 2.1. Gastric Cancer

Gastric cancer is a major cause of cancer-related deaths globally [45]. Substantial evidence indicates that the direct accumulation of MPs, particularly those measuring 0.25 and 1 μm in size, in the gastrointestinal tract enhances cell migration, potentially promoting tumor progression and metastasis [46,47]. Interestingly, MPs have been found to accumulate in the tumor immune microenvironment, where they are associated with a reduction in antitumor cytotoxic cells—such as CD8^+^ T cells, natural killer cells, and dendritic cells—alongside increased neutrophil infiltration in both gastric and pancreatic cancers [46]. However, MPs can also exhibit cytotoxic effects and induce gastric cancer cell death, depending on their intracellular concentration [48] (Figure 2). Studies have shown that PS-MPs at a concentration of 600 mg/L reduce gastric cancer cell viability and stimulate oxidative stress and apoptosis, and that 60 nm PS nanoparticles (PS-NPs) cause more severe DNA damage and promote apoptosis more effectively than 500 nm after 24 h of exposure [48] (Figure 3). Conversely, MP exposure has also been shown to increase the expression of asialoglycoprotein receptor 2 (ASGR2), which is associated with gastric cancer and multidrug resistance to chemotherapy, due to the upregulation of CD44 expression observed in vitro and in vivo after four weeks of exposure [47]. Additionally, sorption experiments have revealed that MPs can act as carriers, transporting polycyclic aromatic hydrocarbons (PAHs) and their derivatives into the human body through oral intake. This increases gastrointestinal toxicity and, consequently, elevates cancer risks in both adults and children [49].

### 2.2. Colorectal Cancer

Colorectal cancer (CRC) is the third most common cancer worldwide, with an increasing incidence in individuals under 50 [50]. Importantly, a dose of 0.1, 1, and 10 μg/mL of PS-MPs smaller than 1 μm enhances cell migration, potentially promoting metastasis in both monolayer and spheroid cultures [51]. Furthermore, a recent study indicated that MP pollutants can mediate protective autophagy through the activation of the mTOR/ULK1 axis, which leads to CRC progression and chemoresistance in vitro and in vivo [52]. Moreover, human colorectal adenocarcinoma Caco-2 and HT-29 cells exposed to micro-sized polyethylene (0.25–1.0 mg/mL) or ethanol-extracted polyethylene for 48 h showed decreased cell viability and increased oxidative stress, particularly mitochondrial superoxide production, in a dose-dependent manner [53]. During early inflammation, MPs induce intestinal inflammation and increase vascular permeability, while long-term ingestion leads to higher serum levels of pro-inflammatory cytokines and lower macrophage aggregation in the gut and brain. In the microbiota–gut–brain axis, MPs can increase inflammation and reactive oxygen species (ROS) levels via activation of the very late antigen 4-vascular cell adhesion molecule 1 (VLA4-VCAM1) pathway, also inducing macrophage reduction in the late phase of inflammation. Specifically, long-term oral ingestion of the smallest MPs (0.1 μm) at a dose of 10 mg/L promoted gut epithelium damage and colitis associated with depressive behaviors in a CRC mouse model after 4 weeks [54]. In addition, MPs altered the gut microbiome of mice and affected the metabolism of carbohydrates and bile acids, thereby exacerbating gut inflammation and damaging the intestinal barrier [54]. Ingestion of MPs from contaminated food in humans correlates positively with fecal MP concentrations and the severity of inflammatory bowel disease (IBD) [55]. Preliminary studies on colectomy specimens obtained from 11 adults have detected MPs within the digestive tract [56]. In the gut microbiota, MPs interact with the gut mucosa and may influence CRC incidence [57]. Specifically, MPs ingested through diet reach the colon and can disrupt the balance between the gut microbiota and the mucus layer, which normally provides a defense against bacteria and toxins. The accumulation of MPs near this mucus layer may facilitate the transfer of carcinogens or the pinocytotic uptake of MPs, exposing cells to carcinogens and triggering signaling pathways that promote inflammation, ultimately compromising barrier function and increasing CRC risk [57] (Figure 3). This suggests that MPs may act as vectors for delivering carcinogenic bacteria (e.g., *E. coli*) in the colon via genotoxin expression [57].

### 2.3. Brain Cancer

CNS tumors, such as glioblastoma, astrocytoma, and neuroblastoma, represent the most aggressive primary brain cancers with the poorest prognosis [58]. The invasive growth and infiltration typical of malignant brain tumors render current therapies, like surgery, radiation, and chemotherapy, largely unsuccessful [59]. Research has demonstrated the neurotoxic effects of MPs at both low (60 μg/mL) and high (240 μg/mL) concentrations, as evidenced by induced oxidative injury, altered blood–brain barrier (BBB) integrity, and suppressed GABA synthesis in the brain. Interestingly, glutathione (GSH) supplementation has been shown to counteract this effect by upregulating GABA levels in the prefrontal cortex and amygdala after MP treatment, both in vitro and in vivo [7]. Exposure to PS-NPs at concentrations ranging from 0.5 to 500 μg/mL has been shown to induce cytotoxicity and mitochondrial dysfunction by affecting complex I in a dose-dependent manner (Figure 3). Specifically, high doses of 500 μg/mL of PS-NPs lead to excessive mitophagy through activation of the AMPK/ULK1 signaling pathway, ultimately causing dopaminergic neuron death [60]. Notably, melatonin at a low dose of 10 mg inhibited PS-NP-induced mitochondrial dysfunction and the AMPK/ULK1 pathway by regulating mitochondrial autophagy in SH-SY5Y cells and dopaminergic neurons of rodent models [60]. Similarly, a high dose of 20 mg/mL causing chronic exposure to MPs significantly increased the proliferative and migratory capacities of U87 glioblastoma cells [62]. Interestingly, at a dose of 0.005 g of MPs, the cells exhibited a tendency to form clusters (spheroids) after a period of 26 days for 72 h in a dose-dependent manner [62].

#### 2.3.1. Nutritional Medicine Mitigates MP Toxicity and Colon Cancer Risk

Nutritional medicine, through functional flavonoids, has recently focused on attenuating damage and toxicity induced by MPs [63]. It is noteworthy that MPs accumulate within the intestine, leading to tissue damage, altered barrier function, and perturbation in the expression of immune response genes and intestinal flora composition. Quercetin has been shown to mitigate MP-induced intestinal damage and immune disorders by reversing intestinal flora imbalances, gene expression changes, and their interactions in mice [63]. Furthermore, cyanidin-3-O-glucoside, a natural anthocyanin derived from red bayberry, attenuated PS-MP-induced colonic inflammation by reversing the increased levels of pro-inflammatory cytokines (IL-6, IL-1β, and TNF-α) and upregulating anti-inflammatory cytokines (IL-22, IL-10, and IL-4). Specifically, PS-MPs significantly increased the abundance of pro-inflammatory bacteria (Desulfovibrio, norank_f_Oscillospiraceae, Helicobacter, and Lachnoclostridium) while decreasing the abundance of anti-inflammatory bacteria (Dubosiella, Akkermansia, and Alistipes), which was reversed after cyanidin-3-O-glucoside treatment. Lastly, metabolomic analysis revealed the amelioration in colonic inflammation through the upregulation of metabolites associated with tryptophan metabolism (e.g., shikimate, L-tryptophan, indole-3-lactic acid, and N-acetylserotonin) and bile acid metabolism (e.g., 3β-hydroxy-5-cholenoic acid, chenodeoxycholate, taurine, and lithocholic acid) after cyanidin-3-O-glucoside administration [64]. Overall, current data highlight the toxic effects of MPs in the intestinal tract, including inflammation, changes in the gut microbiota, and disruption of intestinal barrier function (Figure 3). Conversely, they also emphasize the protective and therapeutic effects of functional nutrients in attenuating and reversing their cellular and molecular damage. However, further research is needed to confirm the chemoprotective potential of nutrients in blocking MP-induced cell damage and to better understand the toxic mechanisms of MPs and their specific relationship with colon cancer risk in humans.

#### 2.3.2. The Nrf2 Pathway in Brain Cancer: The Role of Functional Nutrients

The Nrf2 pathway plays a dual role in chemoprevention and cancer cell proliferation [40,41]. In healthy cells under physiological conditions, the Keap1 protein inhibits Nrf2 activation, leading to its ubiquitination and proteasomal degradation. However, low doses of reactive oxygen species (ROS) and polyphenols can oxidize cysteine residues in Keap1, causing Nrf2 to dissociate from Keap1, followed by Nrf2 stabilization via phosphorylation. Following exposure, Nrf2 translocates into the nucleus, binds to antioxidant response elements (AREs) with the small Maf transcription factor, and initiates transcription and moderate expression of resilience target genes, including Hsp70, HO-1, γ-GCS, Trx, and sirtuins. This cellular mechanism induces cancer chemoprevention, immune surveillance, detoxification, and antioxidation for stress adaptation, cross-tolerance, and brain resilience [41]. However, Nrf2 deregulation due to mutations after MP exposure leads to nuclear accumulation and constitutive activation in various cancer cell lines, promoting drug chemoresistance, immune defects, metabolic reprogramming, cancer growth, and metastasis [41]. This aligns with findings by Almeida et al., who demonstrated that constitutive Nrf2 hyperactivation in temozolomide-resistant glioblastoma multiforme tumors attenuated oxidative stress by increasing SOD and catalase expression [61].

In functional nutrient-based chemoprevention, a study showed the anticancer role of FTY720, a synthetic compound derived from the Isaria sinclairii metabolite, in inhibiting the Nrf2 pathway and its downstream HO-1 and NQO-1 genes, sensitizing human glioblastoma cells to temozolomide [65]. Moreover, FTY720 also induced cell death, autophagy, apoptosis, and necroptosis via Nrf2 suppression, representing a promising therapeutic agent, especially for cancers with constitutive Nrf2 activation like glioblastoma [66]. Therefore, flavonoids can act as antioxidants or pro-oxidants depending on concentration and microenvironment. Low/non-cytotoxic polyphenol concentrations upregulate the Nrf2 antioxidant pathway for cancer chemoprevention, while high concentrations can act as pro-oxidants, downregulating Nrf2 expression and related resilience genes and proteins (e.g., HO-1, Hsp70) by inducing cytotoxic activity in brain cancer cells, suggesting their potential as chemotherapeutic drugs [41]. To date, few studies have explored the role of MP-induced brain cancer. However, recent data suggest that functional nutrients can act as pro- or anti-oncogenic signaling factors in a dose-dependent manner. Interestingly, nutrients block MP-induced oxidative stress and potential cancer risk, potentially leading to new clinical strategies from personalized nutritional therapy to precision medicine for brain tumor prevention and management.

## 3. Functional Nutrition Targeting Stress Resilience Signaling Exerts Anticancer Effects to Mitigate MP Damage

**Figure 4:**
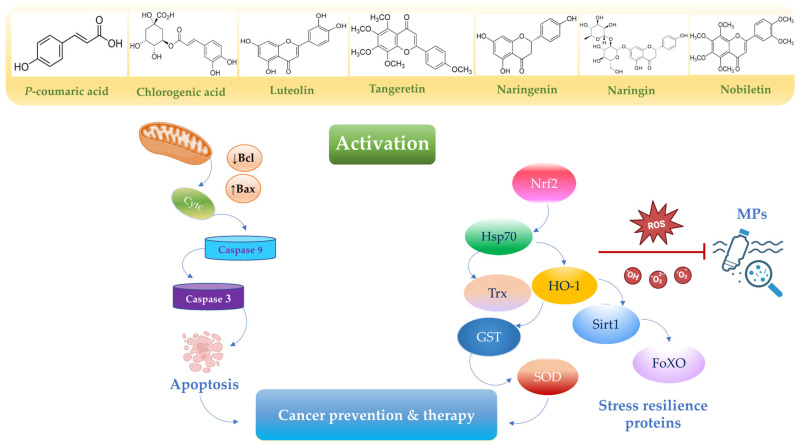
A schematic representation of apoptotic and antioxidant pathways upregulated by functional nutrients. Activation of various signaling cascades, including the Nrf2 pathway and the caspase-9/caspase-3 pathways, contributes to cancer prevention and therapy [73,74,75]. Dysregulated or constitutive activation of Nrf2 signaling due to MP exposure may promote the cancerous transformation of normal cells. Created in BioRender. Scuto, M. (2025); https://BioRender.com/37fzw8x (accessed on 12 May 2025).

**Figure 5:**
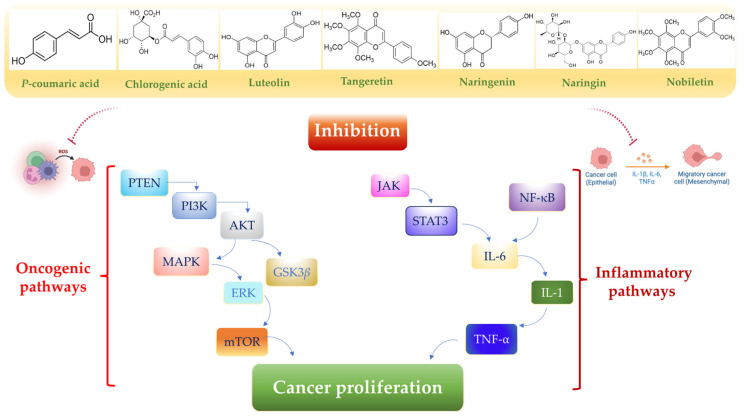
A schematic representation of oncogenic and inflammatory pathways downregulated by functional nutrients. Exposure to MPs activates multiple pro-inflammatory signaling pathways, particularly PTEN, mitogen-activated protein kinase (MAPK), nuclear factor kappa-light-chain-enhancer of activated B cells (NF-κB), and AKT pathways. This activation leads to the release of cytokines such as interleukin-1 (IL-1) and interleukin-6 (IL-6) [73,95,96,97]. Created in BioRender. Scuto, M. (2025); https://BioRender.com/n89f08o (accessed on 12 May 2025).

Nutrition exerts a profound influence on human health and disease progression [67]. Functional nutrients, particularly flavonoids such as chlorogenic acid, *p*-coumaric acid, nobiletin, naringin, naringenin, luteolin, and polyphenol-based nanoparticles, are widely utilized as drugs and dietary supplements due to their low toxicity and dose-dependent pharmaceutical properties [18]. Importantly, these nutrients have been shown to promote anti-inflammatory [68], gastroprotective [69], neuroprotective [70], antioxidant [71], and anticancer properties [72]. The accumulation of MPs in the body tends to trigger free radical overproduction and inflammation, leading to gastric–colon–brain axis dysfunction (Figure 2), which can result in systemic toxicity and cancer development (Figure 3). Currently, the therapeutic potential of functional nutrients in ameliorating MP-induced damage in tissues and organs remains uncertain. However, emerging evidence exploring the therapeutic effects of some flavonoids has resulted in the development of new drug candidates that can mitigate MP-induced oxidative stress and cell proliferation [73] (Figure 4) and (Figure 5).

### 3.1. Chlorogenic Acid

Chlorogenic acid (5-caffeoylquinic acid, CGA), the most abundant phenolic acid in various fruits, vegetables, coffee, and green tea, exhibits promising anticancer properties [74]. Evidence suggests that CGA at 40 μM provides chemoprotective effects by activating the Nrf2 pathway and phase II detoxification enzymes (GST, NQO1) (Figure 4), while inhibiting ROS-mediated NF-κB, MAPK, and AP-1 signaling [75] (Table 2).

**Table 2:** Molecular pathways and protective mechanisms upregulated or downregulated.

Nutrients	Pathways Upregulated	Pathways Downregulated	Outcomes	Ref.
**Chlorogenic acid**	Nrf2, GST, NQO1	NF-κB, MAPK, AP-1	Protect against environmental carcinogen-induced carcinogenesis	[75]
	Bax, caspasi-3	Bcl-2, c-myc, survivin, VEGFA and cyclin D	Induces apoptosis in gastric cancer.	[76,77]
		Cyclin D, Wnt/β-catenin	Inhibits proliferation of A549 human cancer cells and colon cancer.	[78,79,80,81,82]
		TAMs	Reduces tumor growth in a G422 glioma xenograft model.	[83,84,85,86]
	mTOR/TFEB TPK1-PDH	ACAT1	Promotes autophagic flux in neuroblastoma cells	[87,88]
***P*** **-coumaric acid**	Nrf2, HO-1, TRXN, GPX2, GST	NF-κB	Inhibits cell proliferation in gastric adenocarcinoma cells.	[89]
			Inhibits cell proliferation in colon adenocarcinoma and induces apoptosis.	[90,91]
		Cyclin B1, cdc2, mdm2, c-fos, c-jun, c-myc, Bax/Bcl-2 ratio		
			Reduces cell viability by inducing G2/M cell cycle arrest and apoptosis in neuroblastoma N2a and in U87MG glioblastoma cells.	[92,93]
**Luteolin**	Nrf2 NQO1	IL-1, IL-6 JAK/STAT3	Suppresses cell proliferation in colon adenocarcinoma.	[94,95,96,97]
		PI3K/Akt	Inhibits cell proliferation in gastric cancer.	[98,99]
		HDAC	Inhibits cell proliferation in colorectal cancer.	[100,101]
		IL-6/STAT3	Promotes apoptosis in glioblastoma.	[102,103,104,105]
**Tangeretin**	Bax, caspasis-3, caspasis-9		Inhibits cell proliferation in gastric cancer.	[106,107,108,109,110]
	ROS, Bax	JNK	Reduces mitochondrial membrane potential and ATPase activity in colorectal cancer cells.	[111,112]
	PTEN	Cyclin-D, cdc-2	Chemopreventive agent in glioblastoma cells.	[113]
		JAK2-STAT3-BCL-2/BCL-xL	Inhibits glioblastoma multiforme cells and induces pro-apoptotic effects.	[114,115]
**Nobiletin**	Nrf2	SREBP1 PI3K/Akt/mTOR	Inhibits gastric cancer cells in a dose-dependent manner.	[116,117,118,119]
		iNOS, HO-1, NQO1	Inhibits colitis-associated colon carcinogenesis in (AOM)/(DSS)-treated mice.	[120]
		AKT/GSK3β/β-catenin cyclin D1 and CDK4 NF-κB	Induces apoptosis in glioblastoma.	[121,122]
**Naringin**	MAPK	PI3K-AKT/Zeb1	Promotes apoptosis in gastric cancer.	[123,124,125]
	p53, caspase-3	PI3K/Akt/mTOR	Induces autophagy in adenocarcinoma cells	[126,127,128,129]
		Bcl-2, PI3K–Akt	Promotes apoptosis in glioblastoma cells.	[130,131]
**Naringenin**	MAPK, Bax, caspase-3, p53, ASK1	Bcl-2, PI3K–Akt, TGF-β/Smad-3, PRDX1, MMP2 and MMP9	Inhibits cell proliferation in gastric and pancreatic cells.	[132,133,134,135,136,137,138]
		IL-6/STAT3	Attenuates colorectal cancer progression in vitro and in vivo.	[139,140]
	AMPK SOD, CAT, GSH, and GSH-Px			
		Hedgehog MMP/ERK/p38	Reduces glioblastoma cell migration and invasion.	[141,142,143,144]

#### 3.1.1. The Potential Effects of Chlorogenic Acid in Gastric Cancer

Recent studies have demonstrated the antimicrobial activity of CGA, alongside other flavonoids from Persea americana seeds, against Helicobacter pylori-induced gastric cancer in vitro and in silico [76]. Furthermore, CGA and related phenolic compounds have been shown to induce apoptosis in gastric cancer cells by upregulating Bax and caspase-3 (Figure 4) while downregulating Bcl-2, c-myc, survivin, VEGFA, and cyclin D [77] (Table 2).

#### 3.1.2. The Potential Effects of Chlorogenic Acid in Colorectal Cancer

Dihydrocaffeic acid, a gut microbiota-derived metabolite of CGA, demonstrates anticancer activity against colon cancer [78,79]. In colon cancer cells (SW480), subtoxic doses of CGA (375 and 750 µg/mL) reduced cyclin D1 expression and inhibited invasion and migration through Wnt/β-catenin pathway suppression [80]. High-dose CGA (2000 µM) also exhibited cytotoxic effects on 3D-cultured HT-29 colon cancer spheroids [81]. Additionally, caffeic acid phenethyl ester effectively inhibited Hsp70 expression via MAPK14 pathway interaction (Table 2) [82].

#### 3.1.3. The Potential Effects of Chlorogenic Acid in Brain Cancer

Recent preclinical and clinical investigations have highlighted CGA’s protective effects against oxidative stress-associated neurological disorders and cancers [83,84]. CGA has demonstrated neuroprotective effects against cognitive injury by promoting autophagic flux in SH-SY5Y cells and APP/PS1 mice through mTOR/TFEB signaling activation (Table 2) [83]. Moreover, Xue et al. demonstrated that CGA (0.1–1 μM) inhibited IL4-induced macrophage polarization by modulating STAT1 and STAT6 signaling in a dose-dependent manner. In a G422 glioma xenograft model, CGA (20 and 40 mg/kg) reduced tumor growth, correlating with decreased M2-like and increased M1-like tumor-associated macrophages (TAMs) [85]. In glioblastoma, combination therapy of temozolomide (10 mg), chloroquine (10 mg), naringenin (10 mg), and phloroglucinol (50 mg) effectively inhibited malignant glioma proliferation through WNT/β-catenin signaling suppression and induced apoptosis in xenograft models (Table 2) [86]. Metabolomic analysis revealed that thiamine metabolism is implicated in neuroblastoma cell differentiation. CGA (20 and 40 mg/kg) exhibited antitumor activity against neuroblastoma by inhibiting ACAT1 and activating the TPK1-PDH pathway in SH-SY5Y cells and mouse xenograft models [87]. Finally, a clinical trial performed by Kang et al. demonstrated that intramuscular CGA injections (5.5 mg/kg) combined with temozolomide (TMZ) reduced tumor lesion size in grade 4 glioma patients without adverse effects [84]. Studies on CGA show adverse effects at high doses, particularly in Wistar rats. Administration of high doses led to inflammatory reactions and oxidative stress damage, with increases in levels of interleukin-6 (IL-6) and malondialdehyde (MDA), two markers of inflammation and oxidative damage. These effects are particularly evident from the dose of 240 mg/kg, indicating that high doses can alter liver biochemistry, suggesting a potential hepatotoxic effect. Therefore, it is important to exercise caution when using CGA at high doses to avoid potential adverse effects [88]. Most of the research on CGA has been conducted in vitro or in vivo. Clinical studies on humans are still insufficient in number, sample size, and duration of follow-up.

### 3.2. P-Coumaric Acid

*P*-Coumaric acid (*p*-CA), a 4-hydroxycinnamic acid phenolic compound, is widely distributed in plants and mushrooms, existing in both free and bound forms. It exhibits antioxidant, anti-inflammatory, and anticancer properties.

#### 3.2.1. The Potential Effects of *P*-Coumaric Acid in Gastric Cancer

Biosynthesized via the shikimate pathway from phenylalanine and tyrosine, *p*-CA plays a pivotal role in secondary metabolism, serving as a precursor for phenolic acids, flavonoids, lignin, and other secondary metabolites. This suggests its potential as a natural preventive agent against gastric cancer. Emerging evidence indicates that *p*-CA and its derivatives, including kaempferol, astragalin, and tiliroside, at concentrations of 80 and 160 µM, inhibit T antigen formation and NF-κB signaling (Table 2), which are implicated in cell proliferation, death, invasion, and metastasis in gastric adenocarcinoma cells [89].

#### 3.2.2. The Potential Effects of *P*-Coumaric Acid in Colorectal Cancer

Recently, Sharma et al. demonstrated that *p*-CA (100 mg/kg) suppressed colonic preneoplastic lesion formation and reduced polyp incidence in rodent models by scavenging free radicals, exhibiting strong antioxidant and chemoprotective effects in a dose-dependent manner [90]. In a follow-up study, the same authors reported that p-coumaric acid supplementation (100 mg/kg) downregulated colonic proteins involved in cell proliferation (cyclin B1, cdc2, mdm2, c-fos, c-jun, c-myc), altered the Bax/Bcl-2 ratio to induce apoptosis, and upregulated the Nrf2 pathway (Table 2) and its downstream target genes (HO-1, TRXN, GPX2, GST) (Figure 4) in 1,2-dimethylhydrazine-treated rats after 15 weeks [91].

#### 3.2.3. The Potential Effects of *P*-Coumaric Acid in Brain Cancer

In brain tumor studies, low doses of *p*-CA (0.5 and 1 μM) in combination with temozolomide reduced cell viability in U87MG glioblastoma cells by inducing G2/M cell cycle arrest and apoptosis [92]. No protective effect of *p*-CA is explicitly indicated in the literature for the brain. Furthermore, *p*-CA at 150 μmol/L induced significant apoptotic effects in neuroblastoma N2a cells through ROS-mediated cytotoxicity [93]. *p*-CA is generally considered safe at moderate doses, with no documented adverse effects at high doses. Most studies are based on animal models or in vitro cell lines; no clinical data are available in humans.

### 3.3. Luteolin

Luteolin (3,4,5,7-tetrahydroxy flavone), a flavonoid molecule capable of crossing the BBB, exhibits antioxidant, anti-inflammatory, and anticancer properties [94]. It modulates multiple molecular pathways, including JAK-STAT3, Nrf2, mTOR, NF-κB, and TLR signaling (Table 2), and suppresses inflammatory mediators such as IL-1 and IL-6 (Figure 5) [95,96,97].

#### 3.3.1. The Potential Effects of Luteolin in Gastric Cancer

The protective effects of luteolin at the gastric level are expressly indicated in the literature. Accordingly, a recent study demonstrated that luteolin (20 μmol/L) synergistically inhibited gastric cancer cell proliferation and arrested the cell cycle in the S-phase when combined with the LY294002 inhibitor through inhibition of the PI3K/Akt signaling pathway (Table 2) [98]. Conversely, according to Ma et al., a high-dose of luteolin (70 μM) induced apoptosis in gastric cancer cells (HGC-27, MFC, MKN-45) by impairing mitochondrial integrity and function, reducing mitochondrial membrane potential, inhibiting mitochondrial electron transport chain complexes I, III, and V, and increasing ROS generation while decreasing SOD activity [99].

#### 3.3.2. The Potential Effects of Luteolin in Colorectal Cancer

Recent preclinical data indicate that luteolin, at doses ranging from 10 to 60 μM, suppresses colon cancer cell proliferation by upregulating Nrf2 and interacting with p53 in a dose-dependent manner [100]. Similarly, luteolin (7.5, 15, and 30 μM) decreased colon cancer proliferation through epigenetic modifications of the Nrf2 gene and subsequent activation of HO-1 and NQO1 (Table 2) [101]. Notably, luteolin at 15 μM exhibited greater histone deacetylase (HDAC) inhibitory activity than at 30 μM in HCT116 and HT29 cells [101].

#### 3.3.3. The Potential Effects of Luteolin in Brain Cancer

A significant body of evidence has shown that luteolin consistently induces glioblastoma cell apoptosis in a dose-dependent manner [102,103,104,105]. Zong et al. demonstrated that luteolin altered the immune microenvironment by inhibiting IL-6/STAT3 signaling and reducing glioma stem cell aggressiveness (Table 2) [102]. The same study also reported that intracranial luteolin injections (60–120 μM) reduced M2-type macrophage infiltration in intracranial tumor implants [102]. Yuan et al. found that luteolin inhibited glioma cell invasion and migration and promoted apoptosis by increasing Bax and Cyt-c expression [103]. Finally, Lee et al. observed that luteolin (100 and 200 μM) induced apoptosis, while lower doses (50 and 100 μM) promoted autophagy, a mechanism involved in glioma cell survival [104]. Overall, appropriate doses of luteolin, particularly in combination with chemotherapeutic agents, could modulate multiple anticancer pathways, attenuate cell proliferation and metastasis, and improve prognosis in gastric–colon–brain axis cancers. Luteolin is generally considered safe at moderate doses. However, studies at high doses and with prolonged use have shown that it can increase liver enzyme activity in rats—an indicator of liver stress that could suggest long-term adverse effects, especially with chronic use. These effects may manifest as alterations in biochemical parameters and hepatic metabolism, which should be monitored in preclinical and clinical studies. The available data in the literature are derived from in vitro cell models and in vivo animal models; clinical studies in humans are currently lacking.

### 3.4. Tangeretin

Tangeretin, a polymethoxylated flavonoid (PMF) abundant in citrus fruit peels, exhibits anti-proliferative, anti-invasive, anti-metastatic, antioxidant, and neuroprotective activities [106]. Research suggests that polymethoxylated flavonoids are more potent inhibitors of tumor cell growth compared to free hydroxylated flavonoids [107,108,109].

#### 3.4.1. The Potential Effects of Tangeretin in Gastric Cancer

In gastric cancer, tangeretin induces apoptosis through both extrinsic and intrinsic signaling pathways in a dose- and time-dependent manner. Specifically, tangeretin (10–60 μM) activates the p53-mediated intrinsic pathway, leading to mitochondrial apoptosis via upregulation of Bax, caspase-3, and caspase-9 (Figure 4). Additionally, the extrinsic Fas/FasL death receptor pathway interacts with the mitochondrial pathway through caspase-8-mediated cleavage of Bid into tBid, which then translocates to mitochondria and interacts with Bax [110] (Table 2).

#### 3.4.2. The Potential Effects of Tangeretin in Colorectal Cancer

Recently, Yin et al. demonstrated that polymethoxylated flavones reversed drug resistance in colon cancer HCT8/T cells and in a nude mouse model by inhibiting the cellular aerobic glycolysis–ROS–autophagy axis in a dose-dependent manner [111]. Furthermore, tangeretin synergistically combined with 5-fluorouracil significantly reduced mitochondrial membrane potential and ATPase activity while increasing ROS production and apoptosis through c-Jun N-terminal kinase (JNK)-mediated signaling in colorectal cancer cells [112].

#### 3.4.3. The Potential Effects of Tangeretin in Brain Cancer

In glioblastoma, tangeretin (45 μM) increased PTEN expression and decreased the expression of cell cycle regulatory genes (cyclin D, cdc-2) at both transcriptional and translational levels, suggesting its potential as a chemopreventive agent [113]. Chang et al. reported that 5-acetyloxy-6,7,8,4′-tetramethoxyflavone (5-AcTMF), an acetylated tangeretin derivative (50 μM), inhibited glioblastoma multiforme cells and induced pro-apoptotic effects by suppressing the JAK2–STAT3–BCL-2/BCL-xL signaling axis (Table 2) [114]. Despite the lack of clinical trials, preclinical data indicate that tangeretin, either alone or in synergistic combination with chemotherapeutic agents, may be an effective functional supplement for the prevention and mitigation of drug resistance and the management of gastric–colon–brain axis cancers in humans. Tangeretin shows promising therapeutic effects but may have adverse effects at high doses. An acute and subacute toxicity study in mice revealed alterations in clinical and hepatic biochemical profiles, suggesting a potential hepatotoxic effect at high doses, although acute administration up to 3000 mg/kg did not cause fatalities. The dose-dependent relationship is evident, and the sub-lethal hepatic side effects indicate that tangeretin may require long-term monitoring, especially when used in combination with other therapies [115]. Available evidence is limited to preclinical studies; there are no clinical studies in patients.

### 3.5. Nobiletin

Nobiletin (5,6,7,8,3′,4′-hexamethoxyflavone), a polymethoxylated flavone found in Citrus nobilis [116], is characterized by its exclusive presence in citrus fruit peels and exhibits various beneficial activities, including anticancer effects in vitro and in vivo [116,117,118]. Notably, its metabolites also demonstrate anticancer properties [118].

#### 3.5.1. The Potential Effects of Nobiletin in Gastric Cancer

Nobiletin (200 μM) directly targeted sterol regulatory element-binding protein 1 (SREBP1), preventing its nuclear translocation and binding to the ATP citrate lyase (ACLY) promoter. This induced autophagy-dependent cell death through inactivation of the PI3K/Akt/mTOR pathway (Table 2) in a dose-dependent manner in gastric cancer cells [119]. Consistent with these in vitro findings, nobiletin (15–30 mg) inhibited gastric tumor growth, alone and in combination with 5-fluorouracil, in patient-derived xenograft (PDX) models by dose-dependently inhibiting ACLY expression [119].

#### 3.5.2. The Potential Effects of Nobiletin in Colorectal Cancer

Preclinical studies have shown that nobiletin significantly reduced iNOS, HO-1, and NQO1 levels, upregulated Nrf2-dependent enzymes, and modulated key signaling proteins, resulting in the inhibition of colitis-associated colon carcinogenesis in azoxymethane (AOM)/dextran sulfate sodium (DSS)-treated mice [120]. No direct inhibitory effects of nobiletin on colon cancer are explicitly reported in the literature.

#### 3.5.3. The Potential Effects of Nobiletin in Brain Cancer

In glioblastoma, nobiletin (15 μM) suppressed migration and invasion by blocking TGF-β-induced β-catenin nuclear translocation via inhibition of the AKT/GSK3β/β-catenin signaling pathway (Figure 5) [121]. Jiang et al. observed that nobiletin (6, 12, 12.5, and 25 μM) triggered autophagy, evidenced by increased LC3B II and LC3-I expression and decreased p62 expression. It also induced G0/G1 cell cycle arrest, depleted cyclin D1 and CDK4 expression, and suppressed migration and invasion by inhibiting the NF-κB pathway and the pro-inflammatory cytokine cascade in human pancreatic cancer cells (Table 2) [122]. These results suggest that nobiletin holds promise as an anticancer drug candidate for cancer prevention and treatment. However, further studies, particularly toxicological investigations focusing on interactions with emerging pollutants and other flavonoids, are needed to fully understand its potential cancer risk modulation along the gastric–colon–brain axis in humans. Concerning safety, available studies do not report any known adverse effects of nobiletin. Rather, it is consistently described as a promising compound with therapeutic potential, particularly due to its anti-inflammatory and anticancer activities. To date, no toxic effects have been documented; however, further investigation is required to assess its long-term interactions and safety in combination with other substances, especially in complex clinical settings. Currently, evidence is limited to in vitro and animal models, and there is no clinical validation yet.

### 3.6. Naringin

Naringin (4′,5,7-trihydroxy-flavonone-7-rhamnoglucoside), a flavonoid found in grapefruit and citrus fruits, exhibits antioxidant [123], anti-inflammatory [124], and anticancer properties [125].

#### 3.6.1. The Potential Effects of Naringin in Gastric Cancer

Recent studies have shown that cytotoxic doses of naringin (76.21 μM and 64.42 μM) effectively induce cell cycle arrest and apoptosis and inhibit epithelial–mesenchymal transition (EMT) by targeting the PI3K–AKT/Zeb1 pathway in gastric cancer cells (MGC803 and MKN45) [125]. Consistently, naringin (150 mg/kg) significantly reduced tumor growth in BALB/c nude mice [125]. Moreover, naringin (2 μM) induced autophagy and significantly reduced gastric cancer growth by downregulating the PI3K/Akt/mTOR cascade and activating MAPKs (Table 2), suggesting its potential as a natural therapeutic enhancer in adenocarcinoma (Figure 5) [126].

#### 3.6.2. The Potential Effects of Naringin in Colorectal Cancer

Recent evidence demonstrated that naringin (6, 12 or 25 µg/mL) significantly inhibited the proliferation of HCT116 cells in a dose-dependent manner. Notably, naringin promoted the apoptosis of CRC cells and inhibited the activation of the PI3K/AKT/mTOR signaling pathway in a dose-dependent manner [127]. Furthermore, other authors confirmed that naringin (100, 200, and 400 µg/mL) attenuated proliferation and promoted apoptosis dose-dependently in vitro. Similarly, a dose of 50 mg naringin injected intraperitoneally in mice showed an inhibitory effect on tumor growth with good bio-compatibility [128]. Finally, oral administration of 50 and 100 mg/kg of naringin significantly prevented colitis and CRC carcinogenesis by suppressing ER stress-induced autophagy in the colorectal epithelium of mice [129].

#### 3.6.3. The Potential Effects of Naringin in Brain Cancer

No protective effect of naringin is explicitly indicated in the literature for the brain. However, naringin (30 μM) acts as a natural kinase inhibitor by suppressing cancer growth and metastasis through targeting the focal adhesion kinase (FAK) signaling pathway in glioblastoma [130]. Additionally, synergistic treatment with naringin (243 μM) and temozolomide (212.5 μM) induced apoptosis in glioblastoma cells by increasing p53 and caspase-3 levels and decreasing Bcl-2 levels through inhibition of the PI3K/Akt pathway and DNA repair mechanisms (PARP-1 and MGMT) (Figure 4) [131]. This combination also altered metabolomic profiles associated with glioblastoma, targeting the oxidation pathway (fatty acid), metabolism pathways (betaine, methionine, fatty acid, purine, glycerolipid, selenoamino acid, sphingolipid, arginine, proline, glycine, and serine), and biosynthesis pathways (phosphatidylethanolamine, phosphatidylcholine, spermidine, spermine, and carnitine), while increasing sphingosine and ceramide levels [131]. Overall, these findings indicate that naringin, either alone or in combination with chemotherapeutic agents, elicits antitumor responses by targeting multiple signaling pathways. Naringin is considered safe at moderate doses, with no documented adverse effects even at high doses. However, all results are currently limited to in vitro or animal model studies; there are no clinical data available in human populations.

### 3.7. Naringenin

Naringenin (2,3-dihydro-5,7-dihydroxy-2-(4-hydroxyphenyl)-4H-1-benzopyran-4-one), a flavanone extracted from citrus fruits, exhibits potent anti-mutagenic and anti-carcinogenic activities [132]. It inhibits cancer progression through multiple mechanisms, including induction of apoptosis, cell cycle arrest, angiogenesis prevention, and modulation of the Wnt/β-catenin (Table 2), PI3K/Akt, NF-κB, and TGF-β signaling pathways across the gastric–colon–brain axis (Figure 5) [132,133,134,135,136].

#### 3.7.1. The Potential Effects of Naringenin in Gastric Cancer

Network pharmacology studies suggest that naringenin, along with other flavonoids from Citri reticulatae pericarpium–Pinelliae rhizoma, could be used to treat gastric cancer by modulating PI3K–Akt and MAPK signaling pathways (Table 2), thereby regulating tumor cell proliferation, apoptosis, and vascular regeneration [135]. Specifically, naringenin effectively inhibited gastric cancer SGC-7901 cell proliferation, migration, and invasion by downregulating matrix metalloproteinases (MMP2 and MMP9) in a time- and concentration-dependent manner. It also induced pro-apoptotic effects by upregulating Bax and cleaved caspase-3 (Figure 4), while downregulating Bcl-2 and Survivin via inhibition of the AKT signaling pathway [133]. Synergistic treatment with naringenin (40 μM) and ABT-737 (5 μM), a Bcl-2 inhibitor, further promoted apoptosis by upregulating p53 and downregulating AKT in SGC-7901 cells [134]. Notably, the TGF-β1 pathway plays a critical role in chemotherapy drug resistance. In this context, a study showed that naringenin (50 and 100 μM) inhibited pancreatic cancer cell migration and invasion by suppressing TGF-β/Smad-3 signaling and enhanced sensitivity to gemcitabine [136]. Other studies found that combined treatment with naringenin and hesperetin suppressed pancreatic cell migration and inhibited FAK and p38 signaling [137]. Park et al. reported that naringenin (200, 400, and 600 μM) downregulated peroxiredoxin-1 (PRDX1) and activated the apoptosis signal-regulation kinase 1 (ASK1) pathway through ROS production in SNU-213 pancreatic cancer cells [138].

#### 3.7.2. The Potential Effects of Naringenin in Colorectal Cancer

Direct effects of naringenin on colon cancer are reported in the literature [139,140]. Importantly, naringenin (40 mg/kg) in synergy with 5-fluorouracil (12.5 mg/kg) has shown significant effects in inhibiting CRC proliferation, via the activation of the AMPK pathway, to regulate mitochondrial function and induce apoptosis in vitro and in mouse models of CRC [139]. Furthermore, the dose of 100 mg of naringenin improved gut microbiota diversity by increasing the abundance of beneficial bacterial species while reducing opportunistic pathogenic bacteria. In addition, naringenin attenuated high-fat-diet-associated CRC progression by downregulating signaling pathways and upregulating stress resilience pathways including SOD, CAT, GSH, and GSH-Px in C57BL/6 mice [140].

#### 3.7.3. The Potential Effects of Naringenin in Brain Cancer

Naringenin also demonstrates anti-proliferative and anticancer effects in glioblastoma [141,142,143,144]. It exhibits a dual action: at low concentrations (60 μg/mL), it attenuates cell migration, whereas at higher concentrations (114 μg/mL), it significantly reduces Gli-1 and Smo protein expression via inhibition of the Hedgehog signaling pathway in C6 glioblastoma cells [143]. Additionally, Chen et al. reported that naringenin (100–300 μM) reduced glioblastoma cell migration and invasion by blocking the MMPs/ERK/p38 pathway (Table 2) [144]. Preclinical data suggest naringenin’s therapeutic potential in a dose-dependent manner. However, clinical studies investigating its pharmacological role in cancer prevention and treatment are lacking. Future clinical trials are essential to explore the molecular pathways targeted by naringenin in inhibiting cancer proliferation, migration, and invasion across gastric–colon–brain axis tumors in humans. Naringenin is known to have protective effects, with no toxic doses reported in the current literature. Although a large body of in vitro data support its biological activity, the limited number of clinical studies to date has not revealed significant toxicity. Nonetheless, the absence of extensive clinical research limits the ability to draw definitive conclusions regarding the safety and efficacy of long-term therapeutic use in patients. Research data are primarily derived from in vitro studies and animal models; very few studies have been conducted directly in human populations.

## 4. Polyphenol-Based Nanomedicine Platforms Inhibit Gastric–Colon–Brain Axis Cancer

Nanomedicine platforms are increasingly recognized as innovative drug delivery systems for the gastric–colon–brain axis, aimed at preventing or mitigating MP-induced damage [145]. Polyphenol–nanoparticle delivery systems enhance the bioavailability and stability of circulating polyphenols, facilitating their efficient diffusion across the gastric, intestinal, and BBB. This approach holds promise for blocking MP-induced damage and promoting resilience in both chemoprevention and therapy, as demonstrated in in vitro, in vivo, and human studies [138,139,140] (Figure 4).

### 4.1. Polyphenol-Based Nanocarriers in Gastric Cancer

Preclinical evidence indicates that naringenin-loaded nanocarriers (NAR@ZIF-8 liposomes) exhibit sustained drug release and enhanced cytotoxic activity against lung adenocarcinoma (A549) and gastric cancer (SGC-7901) cells compared to free naringenin [146]. Furthermore, Morias et al. have shown that functionalized multi-walled naringenin carbon nanotubes also enhanced cytotoxicity in human alveolar basal epithelium cells while maintaining safety in a human skin cell line (hFB) [147].

### 4.2. Polyphenol-Based Nanocarriers in Colorectal Cancer

Moreover, polymeric nanocarriers encapsulating naringenin, xanthohumol, or isoxanthohumol within pluronic micelles effectively increased the bioavailability and cytotoxicity of xanthohumol and isoxanthohumol in human colon cancer cells compared to single-type micelles [148]. Gold nanoparticles (AuNPs) loaded with high-dose CGA (1000 µM) decreased miR-31 oncogene expression in colon cancer cells [149]. A nano-system combining (50 mg) atorvastatin (50 mg) with RGD-ATST/TAGE CNPs demonstrated significant in vivo anticancer efficacy against colon cancer [150].

### 4.3. Polyphenol-Based Nanocarriers in Brain Cancer

Importantly, Ye et al. found that CGA-encapsulated mannosylated liposomes improved immunotherapeutic efficacy by inhibiting glioma tumor growth and promoting M2-to-M1 macrophage polarization [151]. Additionally, folic acid-modified poly(ethylene glycol)-poly(ε-caprolactone) (Fa-PEG-PCL) nano-micelles loaded with luteolin (3.125 μg/mL, 6.25 μg/mL, and 12.5 μg/mL) promoted apoptosis, inhibited cell proliferation, and suppressed neovascularization more effectively than free luteolin and luteolin/MPEG-PCL in both glioma cells and animal models in a time- and dose-dependent manner [152].

### 4.4. Polyphenol-Based Nanocarriers Inhibit MP-Induced Damage

Recently, environmental pollution caused by plastics has posed a serious challenge to human health. Accordingly, evidence is emerging on how to block plastic-induced toxic damage and disorders [153,154]. A recent study showed that luteolin–graphene oxide nanoparticles have shown promise in mitigating polyethylene terephthalate (PET)-induced neurotoxicity and potential brain cancer risk in zebrafish, primarily through the reduction in oxidative stress and the enhancement in antioxidant defenses [154]. Similarly, quercetin (100 μM) demonstrated neuroprotective effects against polystyrene nanoparticle (PS-NP) exposure in nematodes by downregulating neurodegenerative genes (mec-4, deg-3, unc-68, itr-1, clp-1, asp-3) and upregulating dopamine metabolism genes (cat-2, cat-1, dop-1, dop-2, dop-3) [155]. While the protective role of flavonoids via Nrf2 and MAPK signaling against MP toxicity is increasingly recognized, the application of flavonoid-based nanocarriers in this context remains underexplored. Therefore, further research is imperative to elucidate the underlying molecular mechanisms and validate the therapeutic potential of polyphenol-based nanomedicine for preventing and managing MP-induced damage and associated cancer risks along the gastric–colon–brain axis.

## 5. Other Emerging Contaminants: Perfluoroalkyl and Polyfluoroalkyl Substances (PFASs)

### 5.1. PFAS and Cancer Risk

Perfluoroalkyl and polyfluoroalkyl substances (PFASs), a class of synthetic fluorinated aliphatic compounds, are widely utilized in diverse industrial and consumer applications, including food packaging, fire retardants, and non-stick cookware [156]. Notably, perfluorooctane sulfonate (PFOS), perfluorobutane sulfonic acid (PFBS), perfluorononanoic acid (PFNA), perfluorohexane sulfonic acid (PFHxS), perfluorooctane sulfonamide (PFOSA), and perfluorooctanoic acid (PFOA) exhibit significant environmental persistence, bioaccumulation, and documented human toxicity [157]. While the precise mechanisms and dose–response relationships linking PFAS exposure to cancer risk remain under investigation, positive associations with mutations and cancer development have been observed in both in vitro and in vivo studies [158]. Human exposure to PFASs occurs via multiple pathways, including contaminated drinking water, seafood consumption, inhalation of indoor air, and dermal contact [159]. Epidemiological studies have further demonstrated correlations between PFAS exposure and a range of adverse health effects, including immune and thyroid dysfunction, hepatic disease, metabolic dysregulation, renal and reproductive disorders, and cancer [160]. Recent ecological studies have explored the association between PFAS exposure in drinking water and cancer risk, revealing an increased incidence across various cancer types, including oral cavity/pharynx, lung, digestive system, breast, brain, urinary system, soft tissue, and thyroid [161]. However, a meta-analysis found no association with esophageal, gastric, colorectal, or pancreatic cancers [162].

### 5.2. PFASs and Brain Disorders

The potential adverse effects of PFASs on the nervous system and its functions have also been documented. Specifically, PFASs have the ability to penetrate the BBB and accumulate in the brain, influencing the entry of exogenous compounds. A recent pilot study analyzed 17 target PFASs—perfluorohexanoic acid (PFHxA), PFOA, PFNA, perfluorodecanoic acid (PFDA), PFOS, perfluorooctane sulfonamide (FOSA), and 6:2 Cl-PFESA—in 23 plasma samples from glioma patients that cross the BBB. Among these substances, FOSA showed a strong positive correlation with the development and/or progression of glioma [163]. Early studies demonstrated that PFASs induced neurotoxic effects on brain development. The adverse effects of PFASs on neurodevelopment followed the sequence PFOSA > PFOS > PFBS ≈ PFOA. Notably, PFOS induced oxidative and lipoperoxidative effects by decreasing cell viability at the highest concentration (250 μM) and enhanced expression of the acetylcholine (Ach) phenotype only at 50 μM, displaying an “inverted-U” concentration–effect relationship. PFOA inhibited DNA synthesis only at 250 μM. PFBS produced no effect on DNA synthesis, while PFOSA, at both low and high concentrations, produced significant inhibition of DNA synthesis and triggered elevated toxicity, oxidative stress, and cell loss in both undifferentiated and differentiating cells [164]. More recently, exposure to PFASs at doses of 4.4–80.0 μM induced neurotoxicity and neurobehavioral development in zebrafish larvae [165]. Interestingly, elevated glioma grades were associated with higher concentrations of PFOA, PFOS, and FOSA. Specifically, positive correlations were observed between PFOA concentrations and Ki-67 or P53 expression. These findings suggest that exposure to PFASs may increase the likelihood of developing glioma [166].

### 5.3. PFAS Toxicity: Focus on Functional Nutrients

Recent research has provided evidence regarding the health benefits of functional nutrients in reducing the harmful effects of PFAS absorption and bioaccumulation in humans and experimental models [167,168]. Notably, high PFAS concentrations triggered oxidative stress and toxicity in plants [168] and animals [165]. This led to the activation of the antioxidant response (i.e., ascorbate peroxidase, APX; catalase, CAT; guaiacol peroxidase, POX), and in particular the activity of the detoxifying enzyme GST, to counteract and neutralize increased oxidative stress and ROS generation [168]. Moreover, a cross-sectional clinical study examined serum PFAS compounds in relation to folate concentrations. Notably, the results indicated negative associations between red blood cell folate concentrations and PFOS and PFNA concentrations among adolescents, and between red blood cell folate concentrations and serum PFOA, PFOS, PFNA, and PFHxS concentrations among adults. Therefore, dietary total folate intake was inversely associated with PFAS concentrations [169]. This study elucidates that functional nutrition is essential to prevent or reduce PFAS accumulation in the body and mitigate the adverse health effects. Moreover, Li and colleagues, using metabolomics, have shown that short-chain PFASs tend to accumulate in plant leaves because of their small molecular size and relatively higher water solubility. In particular, amino acids, peptides, fatty acids, and lipids contained in lettuce leaves were downregulated in a dose-dependent manner after PFAS exposure. The metabolism of flavonoids involved the shikimate–phenylpropanoid pathway to cope with the stress caused by PFOA and PFOS. Therefore, plants enhanced the detoxifying response and related pathways after PFAS-induced stress [170]. Overall, future research in this still underexplored field should aim to investigate novel technologies to predict PFAS toxicity in exposed individuals. Finally, we postulate that appropriate nutritional interventions targeting stress resilience pathways and detoxifying enzymes could restore damaged cell membranes and barriers, potentially removing PFAS accumulation in the body and ultimately supporting human health.

## 6. Innovative Technologies to Predict Toxicity and Cancer Risk of Emerging Contaminants: Therapeutic Nutritional Strategies for Future Medicine

Recent efforts have intensified toward developing therapeutic innovations for the detection and elimination of emerging contaminants in biological systems [42,171,172]. The synergistic application of microfluidic platforms and machine learning algorithms offers a promising avenue for advancing personalized and precision medicine in cancer prediction, prevention, and diagnosis. Specifically, the integration of functional nutrients within these platforms may provide a more effective strategy for the targeted eradication of cellular MPs, thereby mitigating associated cancer risks. Table 3 reports the advantages and limitations of each of the techniques used for the detection and quantification of MPs.

### 6.1. Microfluidic Platforms

**Figure 6:**
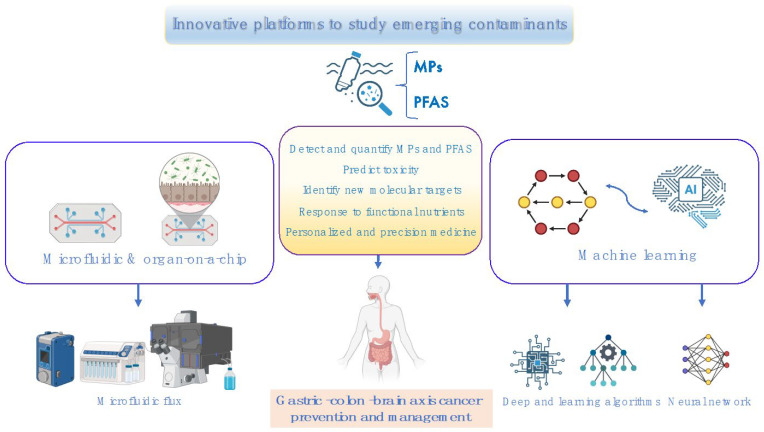
An overview of innovative platforms for studying emerging contaminants. The integration of functional nutrients within these platforms may provide a novel strategy for the targeted eradication of cellular MPs, thereby mitigating associated cancer risks [171,178,186,190,191,199]. Created in BioRender. Scuto, M. (2025); https://BioRender.com/s56suad (accessed on 12 May 2025).

Microfluidic-based detection methods, utilizing diverse biosensing platforms, are increasingly employed for the monitoring and quantification of emerging pollutants, including MPs and PFAS, as well as for investigating their interactions with functional nutrients aimed at mitigating cellular and tissue damage associated with cancer [42,173,174,175,176,177,178] (Figure 6). Recent research has focused on the development of microfluidic devices for the detection, isolation, and separation of MPs in aqueous environments [178]. Among these studies, Faramarzi et al. revealed the efficacy of surface nanodroplet-based microfluidics in capturing small MPs (10 μm in diameter) [179], and the isolation of PS, nylon 6, and polyethylene terephthalate (PET) MPs using Pyrex glass microfluidic systems [180]. Furthermore, microfluidic systems have been utilized to study MP-induced toxicity and neurodegeneration in both in vitro and in vivo models [181,182,183]. The diverse sizes of MPs are a key factor in their toxicity, as smaller particles can penetrate biological barriers and elicit localized effects, including barrier dysfunction, mucus modulation, epithelial cell damage, and interactions with the nervous, immune, and microbiome systems. To assess this, a microfluidic approach was used to examine the impact of PS-MPs, 1 μm in size, and PS-NPs, 100 nm in size, on mouse hippocampal neuronal HT22 cells at concentrations ranging from 5 to 75 μg/mL. The results demonstrated that chronic exposure to smaller-sized PS particles, particularly at higher concentrations, increased ROS production, apoptosis, and S-phase cell cycle arrest, suggesting cytotoxic effects on cellular metabolism and the nervous system, as well as the onset or aggravation of neurodegenerative disorders [181]. Utilizing a microfluidic chip, Liu et al. investigated the interaction of PS-NPs with neurons, varying concentration, surface ligands, and size. Their findings demonstrated that smaller PS-NPs exhibited increased cellular uptake compared to larger particles. Specifically, 80 nm PS-NPs were shown to penetrate and accumulate in the murine brain following aerosol inhalation, resulting in neurotoxicity, evidenced by a reduction in acetylcholinesterase activity compared to water droplet inhalation controls [182]. Furthermore, Youssef et al., employing a microfluidic system, examined the effects of glucose and PS-MPs at concentrations of 100 mg/L and 1000 mg/L on the reproductive function of *Caenorhabditis elegans* (*C. elegans*). Their results revealed that PS MPs at 1000 mg/L significantly reduced egg-laying efficiency and induced a decrease in body size [183]. Beyond the documented effects of MPs on soil-dwelling organisms, microfluidic systems have also been employed to investigate their impact on thrombosis, revealing a significant reduction in fibrin binding to platelets [184]. Notably, Xiao et al. demonstrated that a concentration of 200 μg/mL PS/MPs was internalized into cells via endocytosis and potentially promoted carcinogenic risks by triggering dysregulation of multiple oncogenic signaling pathways. Perturbations were observed in the MAPK signaling pathway (RTK, RAS, ERK, JNK, P38, NRF2, TNF-α, and TNF-α-R) and the PI3K signaling pathway (Figure 5) (PI3K, AKT, MDM2, P53, and BAD) [42]. Collectively, these findings underscore the utility of microfluidic platforms as automated tools for the precise identification and quantification of emerging contaminants within cells and tissues, facilitating the elucidation of molecular pathways perturbed by MP exposure and their potential deleterious effects. The limitations of microfluidics include the high cost and scalability issues for mass production of the necessary components, as well as the complexity of fabrication and operation. In the future, 3D printing will provide the necessary infrastructure for batch production of microfluidic systems capable of sensing and characterizing MPs.

### 6.2. Organ-on-a-Chip Modeling

Organ-on-a-chip platforms, advanced non-invasive in vitro systems, are increasingly utilized to replicate cellular microenvironments for studying disease pathogenesis and for developing novel nutritional therapeutics. The application of microfluidic techniques in nutritional research has expanded, enabling the simulation of whole-body responses to functional nutrients and the detection of health- or disease-associated biomarkers through fluidic channels that mimic physiological interactions. For example, 3D microfluidic devices have been employed to validate the anti-metastatic effects of natural compounds such as sanguinarine, nitidine, and resveratrol [185] (Figure 6). Furthermore, engineered microphysiological models demonstrated that xenohormetic naringenin and soybean-derived glyceollins, at 25 μM, exhibited potent anti-angiogenic effects within the tumor microenvironment in a dose-dependent manner [186]. Microfluidic techniques have also been used to create baicalin liposomes, which exhibited enhanced slow-release properties and stability compared to baicalin monomers, resulting in improved biological activity and bioavailability in zebrafish bioassays [178]. Notably, Lee et al. utilized a multi-organ-on-a-chip system, incorporating liver (HepG2) and tumor (HeLa) cells, to investigate the metabolism-dependent anticancer activity of luteolin, demonstrating its utility in elucidating drug mechanisms involving inter-organ interactions [187]. Collectively, organ-on-a-chip modeling offers a promising approach to assess the protective mechanisms of nutrients in mitigating toxicity and cellular damage, thereby enhancing their therapeutic potential in cancer. The application of this technique supports quantitative extrapolation both in vitro and in vivo (identification and characterization of MPs). Organs-on-chips present multiple potential advantages, including the ability to reproduce the cellular microenvironment under physiological (improved oxygenation and nutrition rates, introduction of shear stress, and improved scaffolds) and pathophysiological (toxicity, stress, and inflammation) conditions. The introduction of additional cell types and tissues from the same or different organs allows for the identification of more complex toxicological mechanisms, assessing toxicity originating from repeated doses/accumulation, size, and the exposure time of emerging contaminants (MPs and PFASs). However, the technology currently faces some limitations, including the need for standardization, the requirement for new or adapted reading systems, challenges with data reproducibility, and the need for trained cell biologists.

### 6.3. Machine Learning Techniques

#### 6.3.1. Machine Learning Devices Predict MP Toxicity

Machine learning techniques are increasingly being employed to quantify environmental contaminants, predict toxicity, identify molecular targets, and personalize dietary interventions for cancer prevention and management [188,189]. The application of artificial neural networks in nutritional science and environmental toxicology presents opportunities for rapid diagnosis, personalized medicine, disease evaluation, and cost-effective healthcare, potentially revolutionizing precision polyphenol-based therapies to mitigate cellular damage from plastic pollutants [190,191] (Figure 6). Notably, machine learning has revealed size-dependent cytotoxicity patterns of MPs in cancer cells, with smaller nanoplastics (≤0.1 μm) exhibiting higher cytotoxicity, likely due to enhanced cellular uptake, which may overwhelm intracellular resilience mechanisms or cause direct damage [190].

#### 6.3.2. Machine Learning Devices Predict Cancer Risk Along the Gastric–Colon–Brain Axis

Preclinical studies have demonstrated the efficacy of machine learning in identifying novel compounds (JFD00950) that inhibit FEN1 activity in colon cancer [190,191]. Quantitative structure–activity relationship (QSAR) modeling has also been used to predict the protective effects of flavonoids in several disorders [192,193]. Interestingly, a clinical study utilizing machine learning has highlighted the importance of antioxidants like naringenin and magnesium in predicting cardiovascular and cancer comorbidities [194]. Machine learning algorithms have also been shown to identify metabolic alterations in the plasma of gastric cancer patients, offering diagnostic and prognostic potential [195]. Additionally, models like the colon oxaliplatin signature (COLOXIS) have been developed to predict responses to oxaliplatin-based regimens [196]. In glioma, cell death-related risk signatures (FANCD2, RRM2, BMP2, NFE2F2, MYD88) have been identified and validated using machine learning [197].

#### 6.3.3. Machine Learning Devices Detect Novel Nutritional Drugs and Targets

Through machine learning clustering analysis, Guo et al. identified licorice flavonoids that interfere with key target proteins in liver cancer, providing new therapeutic drug candidates. The study revealed that seven licochalcone molecules, including glypallichalcone, echinatin, and 3,4,3′,4′-Tetrahydroxy-2-methoxychalcone, interfere with the cancer signaling pathway via the NF-κB signaling pathway, PDL1 expression, and the PD1 checkpoint pathway in cancer. Moreover, the same authors identified the key residues (including ASN364, GLY365, TRP366, and TYR485) involved in the interactions between ten flavonoids and the key target protein (nitric oxide synthase 2) [198]. Machine learning techniques were also used by Cheng et al. to screen and identify the potential target genes significantly upregulated by butein (16 μg/mL), particularly mitogen-activated protein kinase, JAK-STAT, NF-κB, and FoxO in colon cancer cells (Figure 4) [199]. Finally, a multicenter study involving 243 intrahepatic cholangiocarcinoma patients demonstrated that SHAP machine learning effectively identified radiomic signatures of perineural invasion and visualized the prediction process for clinical application [200]. Collectively, machine learning algorithms offer a transformative approach to rapidly identify MP contaminants, predict their cellular and tissue toxicity, and discover novel nutritional targets. This approach facilitates a deeper understanding of the human health consequences, particularly in cancer prevention and therapy.

**Table 3:** Innovative technologies for detection of MPs: advantages and limitations.

Innovative Platforms	MPs Size	Advantages	Limitations	Ref.
Microfluidic platforms (KTP)	50 nm	Simulate the morphology and function of the organs in vitro. Analyze the molecular mechanisms and the damage to cellular micro-structures.	Low ability to capture and identify very small sizes of MPs.	[42]
Surface-nanodroplet-decorated microfluidic device	10 μm	Predicts the adsorption mechanisms and removal of contaminants	Contamination during sample preparation and reduction in capture performance <0.2 μm	[179]
Microfluidic device	PS-MPs, 1 μm PS-NPs, 100 nm	Predicts toxicological and translocation mechanisms of plastic particles and mediate particle–cell interactions.	Does not simulate the shear stress that cells would experience in other physiological microenvironments such as capillary blood flow.	[181]
Microfluidic chip	80 nm	Automatically generates gradient concentrations and rapidly analyzes the interactions between cells and a series of different concentrations of MPs.	Difficult to standardize and scale up. External pumps, tubing, and connectors are required to operate, reducing accessibility and reproducibility and increasing system costs.	[182]
Microfluidic electric parallel egg-laying assay	PS-MPs 1 μm, dose of 100 and 1000 mg/L	Captures and detects different sizes and shapes of MPs using Raman spectroscopy and identifies phenotypical heterogeneity in response to MPs.	Data preprocessing remains complex, making it difficult to detect very small or irregularly shaped particles. Requires extensive sample pre-treatment.	[183]
Microfluidic chip	1 μm	Endothelializes and regionalizes optical irradiation and dynamic blood flow manipulation; realistically reproduces the invasion of MPs.	Requires standardization efforts; reproducibility of data is challenging and trained cell biologists are needed.	[184]
Artificial neural network	0.1–5 mm	Predicts and identifies MP abundance accurately on 67 surface soil samples.	Performance varies depending on dataset quality and spectral noise; preprocessing methods are complex; not suitable for very small or diverse microplastic samples.	[188]
Deep learning algorithms (1D-CNN, SVM AND PLS-DA)	50 µm	Predict the toxicity of MPs and do not require preprocessing of experimental data; can directly analyze and classify spectra with high accuracy.	Difficulty in data collection and lack of standardized criteria. Limited data volume.	[189]
Machine learning device	≤0.1 μm	Enhances predictive accuracy to cytotoxicity in Caco-2 cells.	Diversified datasets and standardized experimental protocols are needed to refine these predictive models.	[190]
Machine learning algorithms (MLR, RF, KNN, SVM, GBDT, AND XGB)	1–25 μm	Improve prediction of MP cytotoxicity with high accuracy; expand the range of MP types that can be detected.	Insufficiency of the number of samples that not adequately represent all possible types of MPs and their diversity. Standardized MPs does not fully capture the complexity of the real environment.	[191]

## 7. Environmental Contaminants, Autism, and Potential Cancer Risk: The Role of Nutrients

### 7.1. MPs and PFASs Enhance the Risk of Developing Autism

Autism Spectrum Disorder (ASD) is a multifaceted neurodevelopmental condition characterized by impairments in social communication, repetitive behaviors, and restricted interests, typically manifesting in early childhood [201]. In recent years, the heightened vulnerability to ASD caused by environmental contaminants, particularly MPs and PFASs, which contribute to brain toxicity, has become a significant global public health concern [202,203]. MPs and PFASs can penetrate the BBB, infiltrating brain tissue and disrupting neuronal function, which can lead to various neurological disorders [163,204]. Recent studies suggest that PFAS exposure during pregnancy may impact neurodevelopment in children. In alignment with this, it has been shown that prenatal exposure to PFOA (1.52–2.17 ng/mL) increases the risk of both ASD and Attention-Deficit Hyperactivity Disorder (ADHD) in children [203]. Specifically, PFASs accumulate in the placenta, cross the placental barrier, and adversely affect fetal development, leading to restricted fetal growth, immune suppression, and neurodevelopmental toxicity [203]. Notably, maternal exposure to PFOS during early development has been linked to embryonic abnormalities. Nrf2a, analogous to the mammalian Nrf2, regulates the embryonic response to PFOS (16 and 32 µM), mitigating oxidative stress, while peroxisome proliferator-activated receptor (PPAR) signaling plays a dose-dependent role in the embryonic adaptive response to PFOS in zebrafish models [205,206]. A recent study explored the effects of PFAS exposure on dopaminergic neurons in *C. elegans*, focusing on morphological changes, synaptic formation, and behavior. Artificial intelligence platforms were utilized to analyze data, providing rapid and accurate results for high-throughput screening of PFAS toxicity. Among the tested PFAS compounds, PFOS (1–100 μmol/L) exhibited significant neurotoxicity, impeding neurodevelopment in *C. elegans* after 48 h of exposure during developmental stages [207]. Similarly, a study by Huang et al. revealed the neurotoxic effects of circulating MPs (5 μg/mL) post-phagocytosis, which obstructed capillaries in the brain, causing thrombosis, neurobehavioral abnormalities, and an increased incidence of ASD [208]. Additionally, research has highlighted the gastrointestinal burden and neurotoxicity of MPs via the gut–brain axis in both zebrafish and humans, acting in a dose-dependent manner [209,210,211,212]. Plastics and chemical contaminants, including MPs and PFASs, function as endocrine-disrupting chemicals (EDCs), disrupting intracellular homeostasis and contributing to brain toxicity, gut dysbiosis, and adverse behavioral outcomes, including ASD [213]

### 7.2. Potential Signaling Pathways Related to Environmental Pollutants, Autism, and Cancer Risk

Emerging evidence suggests a link between pollutants, ASD, and cancer development, particularly along the gastric–colon–brain axis, mediated by PTEN mutations [214,215,216,217]. Under normal conditions, PTEN acts as a tumor suppressor, but somatic mutations in PTEN are common in tumors and in the germline of patients. Notably, germline PTEN mutations in ASD patients correlate with more severe symptoms and an increased cancer risk [216,217,218]. Mechanistic studies have shown that germline PTEN mutations predispose individuals to PTEN hamartoma tumor syndrome (PHTS), a rare inherited cancer syndrome that is also a leading cause of ASD [219]. MPs (1–10 μm) induce oxidative stress, necroptosis, and toxicity by disrupting the PTEN/PI3K/AKT signaling pathway [220] and GSH metabolism [221]. Likewise, PFOS and 6:2 Cl-PFES have been found to activate the PI3K/AKT/mTOR signaling pathway, resulting in microbiota alterations [222] and cancer cell proliferation in vitro and in vivo [223].

### 7.3. Personalized Nutritional Medicine Restores MP and PFAS Damage and Improves Autism

At present, there are no specific pharmacological treatments for ASD. However, nutritional supplementation with functional flavonoids offers promising neuroprotective and therapeutic potential to address the underlying molecular and cellular mechanisms of ASD [224,225,226,227]. In this context, a recent study by Zhang et al. demonstrated that a 40 mg/kg dose of luteolin antagonized LRP1, reducing matrix metallopeptidase-9 (MMP9) expression and leading to significant improvements in autism-like behavior in rat models [224]. Furthermore, combined nutritional interventions with Lactobacillus rhamnosus GG and luteolin notably enhanced the gut microbiome in ASD animal models exposed to the neurotoxic pollutant propionic acid. The study showed that a daily combination of yogurt, L. rhamnosus GG (0.2 mL, 1 × 10^9^ CFU), luteolin (50 mg), and artichokes (400 mg) for 27 days increased antioxidant levels of GPX1 and GSH, as well as GABA signaling in the brain, while reducing pro-inflammatory cytokines such as TNF-α and IL-6 in rodent models of ASD [225]. Thus, functional nutrients exhibit anti-inflammatory and antioxidant effects, making them promising dietary supplements for the prevention and management of ASD. Importantly, disruptions in the PI3K/AKT/mTOR pathway are closely linked to abnormal synaptic protein synthesis and the onset of ASD. In this regard, naringenin significantly inhibited AKT1 phosphorylation, promoted gene transcription and protein synthesis, and activated the Akt-mTOR signaling pathway to prevent apoptosis, alleviating abnormal social behavior in ASD mice models [226,227]. Moreover, lutein-loaded nanoparticles (5 mg/kg) reversed sociability and social memory deficits, as well as anxiety-like and repetitive behaviors induced by prenatal exposure to valproic acid, while restoring oxidative stress markers and apoptosis in the hippocampus of rats [228]. Finally, nose-to-brain delivery of neuro-nutrients has gained significant interest, as it improves absorption, bioavailability, and pharmacological effects for neuropsychiatric disorders [229]. Collectively, we propose that personalized nutritional therapeutics should be considered to mitigate MP- and PFAS-induced oxidative stress and neurotoxicity, potentially restoring disrupted molecular pathways, particularly the PTEN/PI3K/AKT/mTOR axis (Figure 5) and the Nrf2 antioxidant signaling cascade.

## 8. Conclusions and Future Research Perspectives

In conclusion, emerging environmental contaminants pose a significant threat to human health by disrupting metabolic and physiological balance, potentially leading to cancer development along the gastric–colon–brain axis. The persistent accumulation of these pollutants in the environment, coupled with their harmful effects on the human population, is drawing increasing attention from the scientific community. Recent findings suggest that MPs induce systemic genotoxicity and stress by altering various cellular and molecular pathways, including the MAPK and PI3K-AKT-mTOR signaling pathways, thereby promoting carcinogenesis both in vitro and in vivo [44]. Functional nutrition has the potential to mitigate the oxidative stress and toxicity caused by MPs by activating detoxifying Nrf2 resilience pathways and apoptotic mechanisms, which may help prevent and manage cancer along the gastric–colon–brain axis. Notably, functional nutrients such as chlorogenic acid, p-coumaric acid, nobiletin, naringin, naringenin, luteolin, and particularly polyphenol-based nanoparticles with enhanced stability and bioavailability show promise in counteracting MP-induced damage. These nutrients can downregulate carcinogenic signaling pathways, including JAK-STAT3, PI3K/Akt/mTOR, NF-κB, and TLR, while upregulating or restoring the physiological functions of PTEN and Nrf2. However, research into the relationship between emerging contaminants, cancer risk along the gastric–colon–brain axis, and the protective effects of nutrients remains limited and largely unexplored. Therefore, further studies are needed to determine how exposure to different types and sizes of these contaminants may affect the cellular microenvironment, gut microbiota structure, BBB integrity, and overall brain function. While several promising cellular models exist, our review highlights some methodological gaps. Future research should prioritize the development of innovative platforms to assess (i) the duration of MP exposure, the precision of particle sizes, and concentrations; (ii) impairments related to food digestion, nutrient absorption, and transport; (iii) gut microbiota dysbiosis; (iv) inflammatory and immune responses triggered by MPs; and (v) the renewal and regeneration of damaged cells and barriers after nutritional intervention in dose–response relationships. Additionally, long-term cellular models are needed to analyze the toxicological profiles of emerging contaminants, such as MPs and PFAS, especially their persistence and accumulation in human cells and tissues [175,190,230,231]. The bioavailability of circulating MPs or their components, including internalization and migration, should also be explored. To this end, novel platforms, from microfluidic systems to machine learning models, could support long-term investigations of toxicological profiles and the underlying molecular mechanisms of these contaminants. Such platforms should be prioritized in future research efforts. Moreover, the integration of artificial intelligence (AI) and machine learning algorithms to track and quantify individual MP exposure and develop personalized nutritional interventions, possibly in combination with chemotherapeutic agents, presents an unexplored avenue for further investigation. The combination of microfluidic platforms and organ-on-a-chip with AI enables more efficient data acquisition and represents a powerful tool for analyzing emerging contaminants in biological fluids, simultaneously with inter-organ crosstalk. This observation emphasizes the importance of how AI, when applied to these sophisticated devices, can significantly improve precision and efficiency in MP research, accelerating the identification process and surpassing the limitations of conventional techniques, particularly monolayer cell cultures and traditional spectral analysis methodologies. This approach allows for accurate and detailed analysis, enabling statistical evaluation of the samples through the automation of the procedure itself. Further research is crucial to identify and thoroughly investigate the molecular pathways influenced by functional nutrients in preventing or alleviating oxidative stress and cellular toxicity caused by emerging contaminants, as well as their potential role in mitigating cancer risk along the gastric–colon–brain axis. This knowledge will provide a solid scientific foundation for addressing environmental challenges and protecting human health, ultimately paving the way for a therapeutic option based on “personalized and precision medicine”, in the selection of functional nutrients alone and/or in synergy with drugs for patient administration.

## Data Availability

No new data were created or analyzed in this study. Data sharing is not applicable to this article.

## References

[1] Kadac-Czapska K., Knez E., Gierszewska M., Olewnik-Kruszkowska E., Grembecka M. (2023). Microplastics Derived from Food Packaging Waste—Their Origin and Health Risks. Materials.

[2] Li X., Huang Y., Zu D., Liu H., He H., Bao Q., He Y., Liang C., Luo G., Teng Y. (2024). PMMA nanoplastics induce gastric epithelial cellular senescence and cGAS-STING-mediated inflammation via ROS overproduction and NHEJ suppression. Ecotoxicol. Environ. Saf..

[3] Han S.W., Choi J., Ryu K.Y. (2024). Recent Progress and Future Directions of the Research on Nanoplastic-Induced Neurotoxicity. Neural Regen. Res..

[4] Kozlov A.V., Javadov S., Sommer N. (2024). Cellular ROS and Antioxidants: Physiological and Pathological Role. Antioxidants.

[5] Scuto M.C., Mancuso C., Tomasello B., Ontario M., Cavallaro A., Frasca F., Maiolino L., Trovato Salinaro A., Calabrese E.J., Calabrese V. (2019). Curcumin, Hormesis and the Nervous System. Nutrients.

[6] Pitt J.A., Trevisan R., Massarsky A., Kozal J.S., Levin E.D., Di Giulio R.T. (2018). Maternal transfer of nanoplastics to offspring in zebrafish (*Danio rerio*): A case study with nanopolystyrene. Sci. Total Environ..

[7] Yang D., Zhu J., Zhou X., Pan D., Nan S., Yin R., Lei Q., Ma N., Zhu H., Chen J. (2022). Polystyrene micro- and nano-particle co-exposure injures fetal thalamus by inducing ROS-mediated cell apoptosis. Environ. Int..

[8] Manuguerra S., Espinosa Ruiz C., Santulli A., Messina C.M. (2019). Sub-lethal doses of polybrominated diphenyl ethers, in vitro, promote oxidative stress and modulate molecular markers related to cell cycle, antioxidant balance and cellular energy management. Int. J. Environ. Res. Public Health.

[9] Schnee M., Sieler M., Dörnen J., Dittmar T. (2024). Effects of polystyrene nano- and microplastics on human breast epithelial cells and human breast cancer cells. Heliyon.

[10] Chen G., Shan H., Xiong S., Zhao Y., van Gestel C.A.M., Qiu H., Wang Y. (2024). Polystyrene nanoparticle exposure accelerates ovarian cancer development in mice by altering the tumor microenvironment. Sci. Total Environ..

[11] Sung H., Ferlay J., Siegel R.L., Laversanne M., Soerjomataram I., Jermal A., Bray F. (2021). Global cancer statistics 2020: GLOBOCAN estimates of incidence and mortality worldwide for 36 cancers in 185 countries. CA Cancer J. Clin..

[12] Zheng D., Chen L., Tian H., Yang Q., Wu J., Ji Z., Cai J., Chen Y., Li Z. (2022). A scientometric analysis of research trends on emerging contaminants in the field of cancer in 2012–2021. Front. Public Health.

[13] Das A. (2023). The emerging role of microplastics in systemic toxicity: Involvement of reactive oxygen species (ROS). Sci. Total Environ..

[14] Lan Y., Hu L., Feng X., Wang M., Yuan H., Xu H. (2024). Synergistic effect of PS-MPs and Cd on male reproductive toxicity: Ferroptosis via Keap1-Nrf2 pathway. J. Hazard. Mater..

[15] Huang H., Hou J., Yu C., Wei F., Xi B. (2024). Microplastics exacerbate tissue damage and promote carcinogenesis following liver infection in mice. Ecotoxicol. Environ. Saf..

[16] Scuto M., Majzúnová M., Torcitto G., Antonuzzo S., Rampulla F., Di Fatta E., Trovato Salinaro A. (2024). Functional Food Nutrients, Redox Resilience Signaling and Neurosteroids for Brain Health. Int. J. Mol. Sci..

[17] Cordaro M., Salinaro A.T., Siracusa R., D’Amico R., Impellizzeri D., Scuto M., Ontario M.L., Cuzzocrea S., Di Paola R., Fusco R. (2021). Key Mechanisms and Potential Implications of *Hericium erinaceus* in NLRP3 Inflammasome Activation by Reactive Oxygen Species During Alzheimer’s Disease. Antioxidants.

[18] Trovato-Salinaro A., Siracusa R., Di Paola R., Scuto M., Ontario M.L., Bua O., Di Mauro P., Toscano M.A., Petralia C.C., Maiolino L. (2016). Redox modulation of cellular stress response and lipoxin A4 expression by *Hericium Erinaceus* in rat brain: Relevance to Alzheimer’s disease pathogenesis. Immun. Ageing.

[19] Scuto M., Di Mauro P., Ontario M.L., Amato C., Modafferi S., Ciavardelli D., Trovato-Salinaro A., Maiolino L., Calabrese V. (2019). Nutritional Mushroom Treatment in Meniere’s Disease with *Coriolus versicolor*: A Rationale for Therapeutic Intervention in Neuroinflammation and Antineurodegeneration. Int. J. Mol. Sci..

[20] D’Amico R., Trovato-Salinaro A., Cordaro M., Fusco R., Impellizzeri D., Interdonato L., Scuto M.L., Ontario M., Crea R., Siracusa R. (2021). Hidrox^®^ and chronic cystitis: Biochemical evaluation of inflammation, oxidative stress, and pain. Antioxidants.

[21] Cordaro M., Trovato-Salinaro A., Siracusa R., D’Amico R., Impellizzeri D., Scuto M., Ontario M.L., Crea R., Cuzzocrea S., Di Paola R. (2021). Hidrox^®^ Roles in Neuroprotection: Biochemical Links between Traumatic Brain Injury and Alzheimer’s Disease. Antioxidants.

[22] D’Amico R., Trovato-Salinaro A., Fusco R., Cordaro M., Impellizzeri D., Scuto M., Ontario M.L., Lo Dico G., Cuzzocrea S., Di Paola R. (2021). *Hericium erinaceus* and *Coriolus versicolor* Modulate Molecular and Biochemical Changes after Traumatic Brain Injury. Antioxidants.

[23] Trovato-Salinaro A., Siracusa R., Di Paola R., Scuto M., Fronte V., Koverech G., Luca M., Serra A., Toscano M.A., Petralia A. (2016). Redox modulation of cellular stress response and lipoxin A4 expression by Coriolus versicolor in rat brain: Relevance to Alzheimer’s disease pathogenesis. NeuroToxicology.

[24] Cosentino A., Agafonova A., Modafferi S., Trovato-Salinaro A., Scuto M., Maiolino L., Fritsch T., Calabrese E.J., Lupo G., Anfuso C.D. (2024). Blood-Labyrinth Barrier in Health and Diseases: Effect of Hormetic Nutrients. Antioxid. Redox Signal..

[25] Scuto M., Rampulla F., Reali G.M., Spanò S.M., Trovato-Salinaro A., Calabrese V. (2024). Hormetic Nutrition and Redox Regulation in Gut-Brain Axis Disorders. Antioxidants.

[26] Leri M., Scuto M., Ontario M.L., Calabrese V., Calabrese E.J., Bucciantini M., Stefani M. (2020). Healthy Effects of Plant Polyphenols: Molecular Mechanisms. Int. J. Mol. Sci..

[27] Trovato-Salinaro A., Cornelius C., Koverech G., Koverech A., Scuto M., Lodato F., Fronte V., Muccilli V., Reibaldi M., Longo A. (2014). Cellular stress response, redox status, and vitagenes in glaucoma: A systemic oxidant disorder lin-ked to Alzheimer’s disease. Front. Pharmacol..

[28] Bucciantini M., Leri M., Scuto M., Ontario M., Trovato-Salinaro A., Calabrese E.J., Calabrese V., Stefani M. (2022). Xenohormesis underlyes the anti-aging and healthy properties of olive polyphenols. Mech. Ageing Dev..

[29] Trovato-Salinaro A., Pennisi M., Di Paola R., Scuto M., Crupi R., Cambria M.T., Ontario M.L., Tomasello M., Uva M., Maiolino L. (2018). Neuroinflammation and neurohormesis in the pathogenesis of Alzheimer’s disease and Alzheimer-linked pathologies: Modulation by nutritional mushrooms. Immun. Ageing.

[30] Amara I., Ontario M.L., Scuto M., Lo Dico G.M., Sciuto S., Greco V., Abid-Essefi S., Signorile A., Trovato-Salinaro A., Calabrese V. (2021). *Moringa oleifera* Protects SH-SY5YCells from DEHP-Induced Endoplasmic Reticulum Stress and Apoptosis. Antioxidants.

[31] Fusco R., Trovato-Salinaro A., Siracusa R., D’Amico R., Impellizzeri D., Scuto M., Ontario M.L., Crea R., Cordaro M., Cuzzocrea S. (2021). Hidrox^®^ Counteracts Cyclophosphamide-Induced Male Infertility through NRF2 Pathways in a Mouse Model. Antioxidants.

[32] Amara I., Scuto M., Zappalà A., Ontario M.L., Petralia A., Abid-Essefi S., Maiolino L., Signorile A., Trovato-Salinaro A., Calabrese V. (2020). *Hericium Erinaceus* Prevents DEHP-Induced Mitochondrial Dysfunction and Apoptosis in PC12 Cells. Int. J. Mol. Sci..

[33] Siracusa R., Scuto M., Fusco R., Trovato A., Ontario M.L., Crea R., Di Paola R., Cuzzocrea S., Calabrese V. (2020). Anti-inflammatory and Anti-oxidant Activity of Hidrox^®^ in Rotenone-Induced Parkinson’s Disease in Mice. Antioxidants.

[34] Scuto M., Trovato -Salinaro A., Modafferi S., Polimeni A., Pfeffer T., Weigand T., Calabrese V., Schmitt C.P., Peters V. (2020). Carnosine Activates Cellular Stress Response in Podocytes and Reduces Glycative and Lipoperoxidative Stress. Biomedicines.

[35] Cordaro M., Scuto M., Siracusa R., D’amico R., Peritore A.F., Gugliandolo E., Fusco R., Crupi R., Impellizzeri D., Pozzebon M. (2020). Effect of N-palmitoylethanolamine-oxazoline on comorbid neuropsychiatric disturbance associated with inflammatory bowel disease. FASEB J..

[36] Fusco R., Scuto M., Cordaro M., D’Amico R., Gugliandolo E., Siracusa R., Peritore A.F., Crupi R., Impellizzeri D., Cuzzocrea S. (2019). *N*-Palmitoylethanolamide-Oxazoline Protects Against Middle Cerebral Artery Occlusion Injury in Diabetic Rats by Regulating the SIRT1 Pathway. Int. J. Mol. Sci..

[37] Cordaro M., Siracusa R., Fusco R., D’Amico R., Peritore A.F., Gugliandolo E., Genovese T., Scuto M., Crupi R., Mandalari G. (2020). Cashew (*Anacardium occidentale* L.) Nuts Counteract Oxidative Stress and Inflammation in an Acute Experimental Model of Carrageenan-Induced Paw Edema. Antioxidants.

[38] Ijaz M.U., Rafi Z., Hamza A., Sayed A.A., Albadrani G.M., Al-Ghadi M.Q., Abdel-Daim M.M. (2024). Mitigative potential of kaempferide against polyethylene microplastics induced testicular damage by activating Nrf-2/Keap-1 pathway. Ecotoxicol. Environ. Saf..

[39] Tang Y.C., Chuang Y.J., Chang H.H., Juang S.H., Yen G.C., Chang J.Y., Kuo C.C. (2023). How to deal with frenemy NRF2: Targeting NRF2 for chemoprevention and cancer therapy. J. Food Drug Anal..

[40] Scuto M., Ontario M.L., Trovato-Salinaro A., Caligiuri I., Rampulla F., Zimbone V., Modafferi S., Rizzolio F., Canzonieri V., Calabrese E.J. (2022). Redox modulation by plant polyphenols targeting *vitagenes* for chemoprevention and therapy: Relevance to novel anti-cancer interventions and mini-brain organoid technology. Free. Radic. Biol. Med..

[41] Scuto M., Trovato-Salinaro A., Caligiuri I., Ontario M.L., Greco V., Sciuto N., Crea R., Calabrese E.J., Rizzolio F., Canzonieri V. (2021). Redox modulation of *vitagenes* via plant polyphenols and vitamin D: Novel insights for chemoprevention and therapeutic interventions based on organoid technology. Mech. Ageing Dev..

[42] Xiao M., Li X., Zhang X., Duan X., Lin H., Liu S., Sui G. (2023). Assessment of cancer-related signaling pathways in responses to polystyrene nanoplastics via a kidney-testis microfluidic platform (KTP). Sci. Total Environ..

[43] Bruno A., Dovizio M., Milillo C., Aruffo E., Pesce M., Gatta M., Chiacchiaretta P., Di Carlo P., Ballerini P. (2024). Orally Ingested Micro- and Nano-Plastics: A Hidden Driver of Inflammatory Bowel Disease and Colorectal Cancer. Cancers.

[44] Dzierżyński E., Gawlik P.J., Puźniak D., Flieger W., Jóźwik K., Teresiński G., Forma A., Wdowiak P., Baj J., Flieger J. (2024). Microplastics in the Human Body: Exposure, Detection, and Risk of Carcinogenesis: A State-of-the-Art Review. Cancers.

[45] Van Cutsem E., Sagaert X., Topal B., Haustermans K., Prenen H. (2016). Gastric cancer. Lancet.

[46] Zhao J., Zhang H., Shi L., Jia Y., Sheng H. (2024). Detection and quantification of microplastics in various types of human tumor tissues. Ecotoxicol. Environ. Saf..

[47] Kim H., Zaheer J., Choi E.J., Kim J.S. (2022). Enhanced ASGR2 by microplastic exposure leads to resistance to therapy in gastric cancer. Theranostics.

[48] Yan X., Zhang Y., Lu Y., He L., Qu J., Zhou C., Hong P., Sun S., Zhao H., Liang Y. (2020). The Complex Toxicity of Tetracycline with Polystyrene Spheres on Gastric Cancer Cells. Int. J. Environ. Res. Public Health.

[49] Hu X., Yu Q., Gatheru-Waigi M., Ling W., Qin C., Wang J., Gao Y. (2022). Microplastics-sorbed phenanthrene and its derivatives are highly bioaccessible and may induce human cancer risks. Environ. Int..

[50] Eng C., Jacome A.A., Agarwal R., Hayat M.H., Byndloss M.X., Holowatyj A.N., Bailey C., Lieu C.H. (2022). A comprehensive framework for early-onset colorectal cancer research. Lancet Oncol..

[51] Brynzak-Schreiber E., Schögl E., Bapp C., Cseh K., Kopatz V., Jakupec M.A., Weber A., Lange T., Toca-Herrera J.L., Del Favero G. (2024). Microplastics role in cell migration and distribution during cancer cell division. Chemosphere.

[52] Pan W., Han Y., Zhang M., Zhu K., Yang Z., Qiu M., Guo Y., Dong Z., Hao J., Zhang X. (2025). Effects of microplastics on chemo-resistance and tumorigenesis of colorectal cancer. Apoptosis.

[53] Herrala M., Huovinen M., Järvel E., Hellman J., Tolonen P., Lahtela-Kakkonen M., Rysä J. (2023). Micro-sized polyethylene particles affect cell viability and oxidative stress responses in human colorectal adenocarcinoma Caco-2 and HT-29 cells. Sci. Total Environ..

[54] Kuai Y., Chen Z., Xie K., Chen J., He J., Gao J., Yu C. (2024). Long-term exposure to polystyrene microplastics reduces macrophages and affects the microbiota-gut-brain axis in mice. Toxicology.

[55] Yan Z., Liu Y., Zhang T., Zhang F., Ren H., Zhang Y. (2022). Analysis of microplastics in human feces reveals a correlation between fecal microplastics and inflammatory bowel disease status. Environ. Sci. Technol..

[56] Ibrahim Y.S., Tuan Anuar S., Azmi A.A., Wan-Mohd-Khalik W.M.A., Lehata S., Hamzah S.R., Ismail D., Ma Z.F., Dzulkarnaen A., Zakaria Z. (2020). Detection of microplastics in human colectomy specimens. JGH Open.

[57] Li S., Keenan J.I., Shaw I.C., Frizelle F.A. (2023). Could Microplastics Be a Driver for Early Onset Colorectal Cancer?. Cancers.

[58] Louis D.N., Perry A., Wesseling P., Brat D.J., Cree I.A., Figarella-Branger D., Hawkins C., Ng H.K., Pfister S.M., Reifenberger G. (2021). The 2021 WHO Classification of Tumors of the Central Nervous System: A summary. Neuro-Oncology.

[59] Ratliff M., Karimian-Jazi K., Hoffmann D.C., Rauschenbach L., Simon M., Hai L., Mandelbaum H., Schubert M.C., Kessler T., Uhlig S. (2023). Individual glioblastoma cells harbor both proliferative and invasive capabilities during tumor progression. Neuro-Oncol..

[60] Huang Y., Liang B., Li Z., Zhong Y., Wang B., Zhang B., Du J., Ye R., Xian H., Min W. (2023). Polystyrene nanoplastic exposure induces excessive mitophagy by activating AMPK/ULK1 pathway in differentiated SH-SY5Y cells and dopaminergic neurons in vivo. Part. Fibre Toxicol..

[61] Almeida Lima K., Osawa I.Y.A., Ramalho M.C.C., de Souza I., Guedes C.B., Souza Filho C.H.D., Monteiro L.K.S., Latancia M.T., Rocha C.R.R. (2023). Temozolomide Resistance in Glioblastoma by NRF2: Protecting the Evil. Biomedicines.

[62] Rafazi P., Bagheri Z., Haghi-Aminjan H., Rahimifard M., Ahvaraki A. (2024). Long-term exposure of human U87 glioblastoma cells to polyethylene microplastics: Investigating the potential cancer progression. Toxicol. Rep..

[63] Zhao L., Zheng J., Gu Y., Xu X., Yu J., Li J., Yang S., Chen B., Du J., Dong R. (2024). Quercetin intervention mitigates small intestinal damage and immunologic derangement induced by polystyrene nanoplastics: Insights from multi-omics analysis in mice. Environ. Pollut..

[64] Chen W., Zheng X., Yan F., Xu L., Ye X. (2024). Modulation of Gut Microbial Metabolism by Cyanidin-3-*O*-Glucoside in Mitigating Polystyrene-Induced Colonic Inflammation: Insights from 16S rRNA Sequencing and Metabolomics. J. Agric. Food Chem..

[65] Zhang L., Wang H., Zhu J., Ding K., Xu J. (2014). FTY720 reduces migration and invasion of human glioblastoma cell lines via inhibiting the PI3K/AKT/mTOR/p70S6K signaling pathway. Tumor Biol..

[66] Zhang L., Wang H. (2017). FTY720 inhibits the Nrf2/ARE pathway in human glioblastoma cell lines and sensitizes glioblastoma cells to temozolomide. Pharmacol. Rep..

[67] Xiao Y.L., Gong Y., Qi Y.J., Shao Z.M., Jiang Y.Z. (2024). Effects of dietary intervention on human diseases: Molecular mechanisms and therapeutic potential. Signal Transduct. Target. Ther..

[68] Gu T., Zhang Z., Liu J., Chen L., Tian Y., Xu W., Zeng T., Wu W., Lu L. (2023). Chlorogenic Acid Alleviates LPS-Induced Inflammation and Oxidative Stress by Modulating CD36/AMPK/PGC-1α in RAW264.7 Macrophages. Int. J. Mol. Sci..

[69] Truzzi F., Tibaldi C., Zhang Y., Dinelli G.D., Amen E. (2021). An Overview on Dietary Polyphenols and Their Biopharmaceutical Classification System (BCS). Int. J. Mol. Sci..

[70] Sanjay, Sood R., Jaiswal V., Kang S.U., Park M., Lee H.J. (2024). Nobiletin regulates intracellular Ca^2+^ levels via IP_3_R and ameliorates neuroinflammation in Aβ42-induced astrocytes. Redox Biol..

[71] Zhang X., Li M., Wu H., Fan W., Zhang J., Su W., Wang Y., Li P. (2022). Naringenin attenuates inflammation, apoptosis, and ferroptosis in silver nanoparticle-induced lung injury through a mechanism associated with Nrf2/HO-1 axis: In vitro and in vivo studies. Life Sci..

[72] Matić I.Z., Mraković A., Rakočević Z., Stoiljković M., Pavlović V.B., Momić T. (2023). Anticancer effect of novel luteolin capped gold nanoparticles selectively cytotoxic towards human cervical adenocarcinoma HeLa cells: An in vitro approach. J. Trace Elem. Med. Biol..

[73] Cui J., Li X., Gan Q., Lu Z., Du Y., Noor I., Wang L., Liu S., Jin B. (2025). Flavonoids Mitigate Nanoplastics Stress in *Ginkgo biloba*. Plant Cell Environ..

[74] Cortez N., Villegas C., Burgos V., Ortiz L., Cabrera-Pardo J.R., Paz C. (2024). Therapeutic Potential of Chlorogenic Acid in Chemoresistance and Chemoprotection in Cancer Treatment. Int. J. Mol. Sci..

[75] Feng R., Lu Y., Bowman L.L., Qian Y., Castranova V., Ding M. (2005). Inhibition of activator protein-1, NF-kappaB, and MAPKs and induction of phase 2 detoxifying enzyme activity by chlorogenic acid. J. Biol. Chem..

[76] Sánchez-Quezada V., Velázquez-Guadarrama N., Mendoza-Elizalde S., Hernández-Iturriaga M., Landaverdem P.V., Loarca-Piña G. (2024). Bioaccessibility of bioactive compounds present in Persea americana Mill. seed ingredient during oral-gastric digestion with antibacterial capacity against Helicobacter pylori. J. Ethnopharmacol..

[77] Jafari N., Zargar S.J., Delnavazi M.R., Yassa N. (2018). Cell Cycle Arrest and Apoptosis Induction of Phloroacetophenone Glycosides and Caffeoylquinic Acid Derivatives in Gastric Adenocarcinoma (AGS) Cells. Anti-Cancer Agents Med. Chem..

[78] Santana-Gálvez J., Villela-Castrejón J., Serna-Saldívar S.O., Cisneros-Zevallos L., Jacobo-Velázquez D.A. (2020). Synergistic combinations of curcumin, sulforaphane, and dihydrocaffeic acid against human colon cancer cells. Int. J. Mol. Sci..

[79] Santana-Gálvez J., Castrejón J.V., Serna-Saldívar S.O., Jacobo-Velázquez D.A. (2020). Anticancer potential of dihydrocaffeic acid: A chlorogenic acid metabolite. CyTA J. Food.

[80] Villota H., Santa-González G.A., Uribe D., Henao I.C., Arroyave-Ospina J.C., Barrera-Causil C.J., Pedroza-Díaz J. (2022). Modulatory Effect of Chlorogenic Acid and Coffee Extracts on Wnt/β-Catenin Pathway in Colorectal Cancer Cells. Nutrients.

[81] Vélez M.D., Pedroza-Díaz J., Santa-González G.A. (2023). Data on the cytotoxicity of chlorogenic acid in 3D cultures of HT-29 cells. Data Brief.

[82] Yahya S., Sulaiman M.K., Sudhandiran G. (2024). Caffeic acid phenethyl ester mediates apoptosis in serum-starved HT29 colon cancer cells through modulation of heat shock proteins and MAPK pathways. Cell Biochem. Funct..

[83] Gao L., Li X., Meng S., Ma T., Wan L., Xu S. (2020). Chlorogenic Acid Alleviates Aβ_25-35_-Induced Autophagy and Cognitive Impairment via the mTOR/TFEB Signaling Pathway. Drug Des. Dev. Ther..

[84] Kang Z., Li S., Kang X., Deng J., Yang H., Chen F., Jiang J., Zhang J., Li W. (2023). Phase I study of chlorogenic acid injection for recurrent high-grade glioma with long-term follow-up. Cancer Biol. Med..

[85] Xue N., Zhou Q., Ji M., Jin J., Lai F., Chen J., Zhang M., Jia J., Yang H., Zhang J. (2017). Chlorogenic acid inhibits glioblastoma growth through repolarizating macrophage from M2 to M1 phenotype. Sci. Rep..

[86] Daisy Precilla S., Kuduvalli S.S., Biswas I., Bhavani K., Pillai A.B., Thomas J.M., Anitha T.S. (2023). Repurposing synthetic and natural derivatives induces apoptosis in an orthotopic glioma-induced xenograft model by modulating WNT/β-catenin signaling. Fundam. Clin. Pharmacol..

[87] You S., Wang M.J., Hou Z.Y., Wang W.D., Du T.T., Xue N.N., Ji M., Chen X.G. (2023). Chlorogenic Acid Induced Neuroblastoma Cells Differentiation via the ACAT1-TPK1-PDH Pathway. Pharmaceuticals.

[88] Adeyemo-Salami O.A., Afolabi D.A., Amuzat A.A., Adekanye J.O., Oladokun O.O. (2025). Effect of Acute Exposure of Swiss Mice to Chlorogenic Acid. Basic Clin. Pharmacol. Toxicol..

[89] Radziejewska I., Supruniuk K., Tomczyk M., Izdebska W., Borzym-Kluczyk M., Bielawska A., Bielawski K., Galicka A. (2022). *p*-Coumaric acid, Kaempferol, Astragalin and Tiliroside Influence the Expression of Glycoforms in AGS Gastric Cancer Cells. Int. J. Mol. Sci..

[90] Sharma S.H., Chellappan D.R., Chinnaswamy P., Nagarajan S. (2017). Protective effect of p-coumaric acid against 1,2 dimethylhydrazine induced colonic preneoplastic lesions in experimental rats. Biomed. Pharmacother..

[91] Sharma S.H., Rajamanickam V., Nagarajan S. (2019). Supplementation of p-coumaric acid exhibits chemopreventive effect via induction of Nrf2 in a short-term preclinical model of colon cancer. Eur. J. Cancer Prev..

[92] Oliva M.A., Castaldo S., Rotondo R., Staffieri S., Sanchez M., Arcella A. (2022). Inhibiting effect of *p*-Coumaric acid on U87MG human glioblastoma cell growth. J. Chemother..

[93] Shailasree S., Venkataramana M., Niranjana S.R., Prakash H.S. (2015). Cytotoxic effect of p-Coumaric acid on neuroblastoma, N2a cell via generation of reactive oxygen species leading to dysfunction of mitochondria inducing apoptosis and autophagy. Mol. Neurobiol..

[94] Anson D.M., Wilcox R.M., Huseman E.D., Stump T.A., Paris R.L., Darkwah B.O., Lin S., Adegoke A.O., Gryka R.J., Jean-Louis D.S. (2018). Luteolin Decreases Epidermal Growth Factor Receptor-Mediated Cell Proliferation and Induces Apoptosis in Glioblastoma Cell Lines. Basic Clin. Pharmacol. Toxicol..

[95] Farooqi A.A., Butt G., El-Zahaby S.A., Attar R., Sabitaliyevich U.Y., Jovic J.J., Tang K.F., Naureen H., Xu B. (2020). Luteolin mediated targeting of protein network and microRNAs in different cancers: Focus on JAK-STAT, NOTCH, mTOR and TRAIL-mediated signaling pathways. Pharmacol. Res..

[96] De Stefano A., Caporali S., Di Daniele N., Rovella V., Cardillo C., Schinzari F., Minieri M., Pieri M., Candi E., Bernardini S. (2021). Anti-Inflammatory and Proliferative Properties of Luteolin-7-O-Glucoside. Int. J. Mol. Sci..

[97] Jiang J., Zhu F., Zhang H., Sun T., Fu F., Chen X., Zhang Y. (2022). Luteolin suppresses the growth of colon cancer cells by inhibiting the IL-6/STAT3 signaling pathway. J. Gastrointest. Oncol..

[98] Yajie D., Feng L., Zhaoyan L.I., Yan X., Nida C., Guangao Z., Rui W., Aiguang Z. (2023). Efficacy of luteolin on the human gastric cancer cell line MKN45 and underlying mechanism. J. Tradit. Chin. Med..

[99] Ma J., Pan Z., Du H., Chen X., Zhu X., Hao W., Zheng Q., Tang X. (2023). Luteolin induces apoptosis by impairing mitochondrial function and targeting the intrinsic apoptosis pathway in gastric cancer cells. Oncol. Lett..

[100] Kang K.A., Piao M.J., Hyun Y.J., Zhen A.X., Cho S.J., Ahn M.J., Yi J.M., Hyun J.W. (2019). Luteolin promotes apoptotic cell death via upregulation of Nrf2 expression by DNA demethylase and the interaction of Nrf2 with p53 in human colon cancer cells. Exp. Mol. Med..

[101] Zuo Q., Wu R., Xiao X., Yang C., Yang Y., Wang C., Lin L., Kong A.N. (2018). The dietary flavone luteolin epigenetically activates the Nrf2 pathway and blocks cell transformation in human colorectal cancer HCT116 cells. J. Cell. Biochem..

[102] Zong S., Li X., Zhang G., Hu J., Li H., Guo Z., Zhao X., Chen J., Wang Y., Jing Z. (2024). Effect of luteolin on glioblastoma’s immune microenvironment and tumor growth suppression. Phytomedicine.

[103] Yuan X., Ouyang J., Long C. (2024). Effects and Mechanism of Luteolin on Proliferation and Apoptosis of Glioma. Altern. Ther. Health Med..

[104] Lee H.S., Park B.S., Kang H.M., Kim J.H., Shin S.H., Kim I.R. (2021). Role of Luteolin-Induced Apoptosis and Autophagy in Human Glioblastoma Cell Lines. Medicina.

[105] Pradeep S., Sai Chakith M.R., Sindhushree S.R., Reddy P., Sushmitha E., Purohit M.N., Suresh D., Swamy Shivananju N., Silina E., Manturova N. (2025). Exploring shared therapeutic targets for Alzheimer’s disease and glioblastoma using network pharmacology and protein-protein interaction approach. Front Chem..

[106] Raza W., Luqman S., Meena A. (2020). Prospects of tangeretin as a modulator of cancer targets/pathways. Pharmacol. Res..

[107] Manthey J.A., Guthrie N. (2002). Antiproliferative activities of citrus flavonoids against six human cancer cell lines. J. Agric. Food Chem..

[108] Mdkhana B., Zaher D.M., Abdin S.M., Omar H.A. (2021). Tangeretin boosts the anticancer activity of metformin in breast cancer cells via curbing the energy production. Phytomedicine.

[109] Pereira C.V., Duarte M., Silva P., Bento da Silva A., Duarte C.M.M., Cifuentes A., García-Cañas V., Bronze M.R., Albuquerque C., Serra A.T. (2019). Polymethoxylated Flavones Target Cancer Stemness and Improve the Antiproliferative Effect of 5-Fluorouracil in a 3D Cell Model of Colorectal Cancer. Nutrients.

[110] Dong Y., Cao A., Shi J., Yin P., Wang L., Ji G., Xie J., Wu D. (2014). Tangeretin, a citrus polymethoxyflavonoid, induces apoptosis of human gastric cancer AGS cells through extrinsic and intrinsic signaling pathways. Oncol. Rep..

[111] Yin Y., Wu Y.U., Huang H., Duan Y., Yuan Z., Cao L., Ying J., Zhou Y., Feng S. (2024). The superiority of PMFs on reversing drug resistance of colon cancer and the effect on aerobic glycolysis-ROS-autophagy signaling axis. Oncol. Res..

[112] Dey D.K., Chang S.N., Vadlamudi Y., Park J.G., Kang S.C. (2020). Synergistic therapy with tangeretin and 5-fluorouracil accelerates the ROS/JNK mediated apoptotic pathway in human colorectal cancer cell. Food Chem. Toxicol..

[113] Ma L.L., Wang D.W., Yu X.D., Zhou Y.L. (2016). Tangeretin induces cell cycle arrest and apoptosis through upregulation of PTEN expression in glioma cells. Biomed. Pharmacother..

[114] Cheng Y.P., Li S., Chuang W.L., Li C.H., Chen G.J., Chang C.C., Or C.R., Lin P.Y., Chang C.C. (2019). Blockade of STAT3 Signaling Contributes to Anticancer Effect of 5-Acetyloxy-6,7,8,4′-Tetra-Methoxyflavone, a Tangeretin Derivative, on Human Glioblastoma Multiforme Cells. Int. J. Mol. Sci..

[115] Ting Y., Chiou Y.S., Jiang Y., Pan M.H., Lin Z., Huang Q. (2015). Safety evaluation of tangeretin and the effect of using emulsion-based delivery system: Oral acute and 28-day sub-acute toxicity study using mice. Food Res. Int..

[116] Peng Z., Song L., Chen M., Liu Z., Yuan Z., Wen H., Zhang H., Huang Y., Peng Z., Yang H. (2024). Neofunctionalization of an *OMT* cluster dominates polymethoxyflavone biosynthesis associated with the domestication of citrus. Proc. Natl. Acad. Sci. USA.

[117] Rooprai H.K., Kandanearatchi A., Maidment S.L., Christidou M., Trillo-Pazos G., Dexter D.T., Rucklidge G.J., Widmer W., Pilkington G.J. (2001). Evaluation of the effects of swainsonine, captopril, tangeretin and nobiletin on the biological behaviour of brain tumour cells in vitro. Neuropathol. Appl. Neurobiol..

[118] Wu X., Song M., Gao Z., Sun Y., Wang M., Li F., Zheng J., Xiao H. (2017). Nobiletin and its colonic metabolites suppress colitis-associated colon carcinogenesis by down-regulating iNOS, inducing antioxidative enzymes and arresting cell cycle progression. J. Nutr. Biochem..

[119] Chen M., Li H., Zheng S., Shen J., Chen Y., Li Y., Yuan M., Wu J., Sun Q. (2024). Nobiletin targets SREBP1/ACLY to induce autophagy-dependent cell death of gastric cancer cells through PI3K/Akt/mTOR signaling pathway. Phytomedicine.

[120] Tung Y.C., Chou Y.C., Hung W.L., Cheng A.C., Yu R.C., Ho C.T., Pan M.H. (2019). Polymethoxyflavones: Chemistry and Molecular Mechanisms for Cancer Prevention and Treatment. Curr. Pharmacol. Rep..

[121] Jiang H., Chen H., Jin C., Mo J., Wang H. (2020). Nobiletin flavone inhibits the growth and metastasis of human pancreatic cancer cells via induction of autophagy, G0/G1 cell cycle arrest and inhibition of NF-kB signalling pathway. J. BUON.

[122] Zhang X., Zheng K., Li C., Zhao Y., Li H., Liu X., Long Y., Yao J. (2017). Nobiletin inhibits invasion via inhibiting AKT/GSK3β/β-catenin signaling pathway in Slug-expressing glioma cells. Oncol. Rep..

[123] Amini N., Sarkaki A., Dianat M., Mard S.A., Ahangarpour A., Badavi M. (2019). Protective effects of naringin and trimetazidine on remote effect of acute renal injury on oxidative stress and myocardial injury through Nrf-2 regulation. Pharmacol. Rep..

[124] Al-Aubaidy H.A., Dayan A., Deseo M.A., Itsiopoulos C., Jamil D., Hadi N.R., Thomas C.J. (2021). Twelve-Week Mediterranean Diet Intervention Increases Citrus Bioflavonoid Levels and Reduces Inflammation in People with Type 2 Diabetes Mellitus. Nutrients.

[125] Zhu L., Shi J., Mu M., Chen Z., Zhao C., Li X., Qu C., Ye C., Zhao W., Sun X. (2023). Naringin Inhibits the Proliferation, Migration, Invasion and Epithelial-to-Mesenchymal Transition of Gastric Cancer Cells via the PI3K/AKT Signaling Pathway. Altern. Ther. Health Med..

[126] Raha S., Yumnam S., Hong G.E., Lee H.J., Saralamma V.V., Park H.S., Heo J.D., Lee S.J., Kim E.H., Kim J.A. (2015). Naringin induces autophagy-mediated growth inhibition by downregulating the PI3K/Akt/mTOR cascade via activation of MAPK pathways in AGS cancer cells. Int. J. Oncol..

[127] Cheng H., Jiang X., Zhang Q., Ma J., Cheng R., Yong H., Shi H., Zhou X., Ge L., Gao G. (2020). Naringin inhibits colorectal cancer cell growth by repressing the PI3K/AKT/mTOR signaling pathway. Exp. Ther. Med..

[128] Zeng J.N., Tan J.Y., Mo L. (2023). Naringin Inhibits Colorectal Carcinogenesis by Inhibiting Viability of Colorectal Cancer Cells. Chin. J. Integr. Med..

[129] Zhang Y.S., Wang F., Cui S.X., Qu X.J. (2018). Natural dietary compound naringin prevents azoxymethane/dextran sodium sulfate-induced chronic colorectal inflammation and carcinogenesis in mice. Cancer Biol. Ther..

[130] Li J., Dong Y., Hao G., Wang B., Wang J., Liang Y., Liu Y., Zhen E., Feng D., Liang G. (2017). Naringin suppresses the development of glioblastoma by inhibiting FAK activity. J. Drug Target..

[131] Bisht P., Prasad S.R., Choudhary K., Pandey R., Aishwarya D., Aravind V., Ramalingam P., Velayutham R., Kumar N. (2024). Naringin and temozolomide combination suppressed the growth of glioblastoma cells by promoting cell apoptosis: Network pharmacology, in-vitro assays and metabolomics based study. Front. Pharmacol..

[132] Motallebi M., Bhia M., Rajani H.F., Bhia I., Tabarraei H., Mohammadkhani N., Pereira-Silva M., Kasaii M.S., Nouri-Majd S., Mueller A.L. (2022). Naringenin: A potential flavonoid phytochemical for cancer therapy. Life Sci..

[133] Bao L., Liu F., Guo H.B., Li Y., Tan B.B., Zhang W.X., Peng Y.H. (2016). Naringenin inhibits proliferation, migration, and invasion as well as induces apoptosis of gastric cancer SGC7901 cell line by downregulation of AKT pathway. Tumor Biol..

[134] Zhang H., Zhong X., Zhang X., Shang D., Zhou Y.I., Zhang C. (2016). Enhanced anticancer effect of ABT-737 in combination with naringenin on gastric cancer cells. Exp. Ther. Med..

[135] Song S., Huang W., Lu X., Liu J., Zhou J., Li Y., Shu P. (2021). A Network Pharmacology Study Based on the Mechanism of Citri Reticulatae Pericarpium-Pinelliae Rhizoma in the Treatment of Gastric Cancer. Evid.-Based Complement. Altern. Med..

[136] Lou C., Zhang F., Yang M., Zhao J., Zeng W., Fang X., Zhang Y., Zhang C., Liang W. (2012). Naringenin decreases invasiveness and metastasis by inhibiting TGF beta-induced epithelial to mesenchymal transition in pancreatic cancer cells. PLoS ONE.

[137] Lee J., Kim D.H., Kim J.H. (2019). Combined administration of naringenin and hesperetin with optimal ratio maximizes the anti-cancer effect in human pancreatic cancer via down regulation of FAK and p38 signaling pathway. Phytomedicine.

[138] Park H.J., Choi Y.J., Lee J.H., Nam M.J. (2017). Naringenin causes ASK1-induced apoptosis via reactive oxygen species in human pancreatic cancer cells. Food Chem. Toxicol..

[139] Wang D., Zhou Y., Hua L., Hu M., Zhu N., Liu Y., Zhou Y. (2024). The role of the natural compound naringenin in AMPK-mitochondria modulation and colorectal cancer inhibition. Phytomedicine.

[140] Sun J., Shi L., Xu F., Sun H., Liu Y., Sun J., Zhou Q. (2025). Naringenin Inhibits Colorectal Cancer associated with a High-Fat Diet through Modulation of Gut Microbiota and IL-6/STAT3 Pathway. J. Microbiol. Biotechnol..

[141] Gautam M., Gabrani R. (2022). Combinatorial Effect of Temozolomide and Naringenin in Human Glioblastoma Multiforme Cell Lines. Nutr. Cancer.

[142] Zaim Ö., Doğanlar O., Banu Doğanlar Z., Özcan H., Zreigh M.M., Kurtdere K. (2022). Novel synthesis naringenin-benzyl piperazine derivatives prevent glioblastoma invasion by inhibiting the hypoxia-induced IL6/JAK2/STAT3 axis and activating caspase-dependent apoptosis. Bioorganic Chem..

[143] Sargazi M.L., Juybari K.B., Tarzi M.E., Amirkhosravi A., Nematollahi M.H., Mirzamohammdi S., Mehrbani M., Mehrabani M., Mehrabani M. (2021). Naringenin attenuates cell viability and migration of C6 glioblastoma cell line: A possible role of hedgehog signaling pathway. Mol. Biol. Rep..

[144] Chen Y.Y., Chang Y.M., Wang K.Y., Chen P.N., Hseu Y.C., Chen K.M., Yeh K.T., Chen C.J., Hsu L.S. (2019). Naringenin inhibited migration and invasion of glioblastoma cells through multiple mechanisms. Environ. Toxicol..

[145] Hou Y., Tu S., Zhao X., Li G., Li N., Zou A. (2023). An integrative method for evaluating the biological effects of nanoparticle-protein corona. Biochim. Biophys. Acta Gen. Subj..

[146] Wang Z., Liu L., Yin W., Liu Z., Shi L., Tang M. (2021). A Novel Drug Delivery System: The Encapsulation of Naringenin in Metal-Organic Frameworks into Liposomes. AAPS PharmSciTech.

[147] Morais R.P., Novais G.B., Sangenito L.S., Santos A.L.S., Priefer R., Morsink M., Mendonça M.C., Souto E.B., Severino P., Cardoso J.C. (2020). Naringenin-Functionalized Multi-Walled Carbon Nanotubes: A Potential Approach for Site-Specific Remote-Controlled Anticancer Delivery for the Treatment of Lung Cancer Cells. Int. J. Mol. Sci..

[148] Ronka S., Kowalczyk A., Baczynska D., Zółnierczyk A.K. (2023). Pluronics-Based Drug Delivery Systems for Flavonoids Anticancer Treatment. Gels.

[149] Luque-Badillo A.C., Hernandez-Tapia G., Ramirez-Castillo D.A., Espinoza-Serrano D., Cortes-Limon A.M., Cortes-Gallardo J.P., Jacobo-Velázquez D.A., Martinez-Fierro M.L., Rios-Ibarra C.P. (2021). Gold nanoparticles enhance microRNA 31 detection in colon cancer cells after inhibition with chlorogenic acid. Oncol. Lett..

[150] Bao H., Zheng N., Li Z., Zhi Y. (2020). Synergistic Effect of Tangeretin and Atorvastatin for Colon Cancer Combination Therapy: Targeted Delivery of These Dual Drugs Using RGD Peptide Decorated Nanocarriers. Drug Des. Dev. Ther..

[151] Ye J., Yang Y., Jin J., Ji M., Gao Y., Feng Y., Wang H., Chen X., Liu Y. (2020). Targeted delivery of chlorogenic acid by mannosylated liposomes to effectively promote the polarization of TAMs for the treatment of glioblastoma. Bioact. Mater..

[152] Wu C., Xu Q., Chen X., Liu J. (2019). Delivery luteolin with folacin-modified nanoparticle for glioma therapy. Int. J. Nanomed..

[153] Liu X., Zhang M., Tian Y., Liu R., Wang Y., Guo F., Gong Y., Yan M. (2022). Development, Characterization, and Investigation of In Vivo Targeted Delivery Efficacy of Luteolin-Loaded, Eudragit S100-Coated mPEG-PLGA Nanoparticles. AAPS PharmSciTech.

[154] Guru A., Murugan R., Almutairi B.O., Arokiyaraj S., Arockiaraj J. (2023). Brain targeted luteolin-graphene oxide nanoparticle abrogates polyethylene terephthalate induced altered neurological response in zebrafish. Mol. Biol. Rep..

[155] Lei S., Hu Z., Liu H. (2024). Treatment with quercetin mitigates polystyrene nanoparticle-induced reduction in neuron capacity by inhibiting dopaminergic neurodegeneration and facilitating dopamine metabolism in Caenorhabditis elegans. Chemosphere.

[156] Liu D., Tang B., Nie S., Zhao N., He L., Cui J., Mao W., Jin H. (2023). Distribution of per- and poly-fluoroalkyl substances and their precursors in human blood. J. Hazard. Mater..

[157] Imir O.B., Kaminsky A.Z., Zuo Q.Y., Liu Y.J., Singh R., Spinella M.J., Irudayaraj J., Hu W.Y., Prins G.S., Madak Erdogan Z. (2021). Per- and Polyfluoroalkyl Substance Exposure Combined with High-Fat Diet Supports Prostate Cancer Progression. Nutrients.

[158] Schrenk D., Bignami M., Bodin L., Chipman J.K., del Mazo J., Grasl-Kraupp B., Hogstrand C., Hoogenboom L.R., Leblanc J.C., Nebbia C.S. (2020). Scientific Opinion on the risk to human health related to the presence of perfluoroalkyl substances in food. EFSA J..

[159] Trudel D., Horowitz L., Wormuth M., Scheringer M., Cousins I.T., Hungerbühler K. (2008). Estimating Consumer Exposure to PFOS and PFOA. Risk Anal..

[160] Fenton S.E., Ducatman A., Boobis A., DeWitt J.C., Lau C., Ng C., Smith J.S., Roberts S.M. (2021). Per- and polyfluoroalkyl substance toxicity and human health review: Current state of knowledge and strategies for informing future research. Environ. Toxicol. Chem..

[161] Li S., Oliva P., Zhang L., Goodrich J.A., McConnell R., Conti D.V., Chatzi L., Aung M. (2025). Associations between per-and polyfluoroalkyl substances (PFAS) and county-level cancer incidence between 2016 and 2021 and incident cancer burden attributable to PFAS in drinking water in the United States. J. Expo. Sci. Environ. Epidemiol..

[162] Zhang S., Kappil E.M., Zheng T., Boffetta P., Seyyedsalehi M.S. (2024). Per- and poly-fluoroalkyl substances exposure and risk of gastrointestinal cancers: A systematic review and meta-analysis. Eur. J. Cancer Prev..

[163] Xie M.Y., Lin Z.Y., Sun X.F., Feng J.J., Mai L., Wu C.C., Huang G.L., Wang P., Liu Y.W., Liu L.Y. (2024). Per- and polyfluoroalkyl substances (PFAS) exposure in plasma and their blood-brain barrier transmission efficiency—A pilot study. Environ. Int..

[164] Slotkin T.A., MacKillop E.A., Melnick R.L., Thayer K.A., Seidler F.J. (2008). Developmental neurotoxicity of perfluorinated chemicals modeled in vitro. Environ. Health Perspect..

[165] Gaballah S., Swank A., Sobus J.R., Howey X.M., Schmid J., Catron T., McCord J., Hines E., Strynar M., Tal T. (2020). Evaluation of Developmental Toxicity, Developmental Neurotoxicity, and Tissue Dose in Zebrafish Exposed to GenX and Other PFAS. Environ. Health Perspect..

[166] Xie M.Y., Sun X.F., Wu C.C., Huang G.L., Wang P., Lin Z.Y., Liu Y.W., Liu L.Y., Zeng E.Y. (2023). Glioma is associated with exposure to legacy and alternative per- and polyfluoroalkyl substances. J. Hazard. Mater..

[167] Sun Q., Wang T., Zhan X., Hong S., Lin L., Tan P., Xiong Y., Zhao H., Zheng Z., Bi R. (2023). Legacy and novel perfluoroalkyl substances in raw and cooked squids: Perspective from health risks and nutrient benefits. Environ. Int..

[168] Pietrini F., Wyrwicka-Drewniak A., Passatore L., Nogués I., Zacchini M., Donati E. (2024). PFOA accumulation in the leaves of basil (*Ocimum basilicum* L.) and its effects on plant growth, oxidative status, and photosynthetic performance. BMC Plant Biol..

[169] Zhang Y., Mustieles V., Wang Y.X., Sun Y., Agudelo J., Bibi Z., Torres N., Oulhote Y., Slitt A., Messerlian C. (2023). Folate concentrations and serum perfluoroalkyl and polyfluoroalkyl substance concentrations in adolescents and adults in the USA (National Health and Nutrition Examination Study 2003–2016): An observational study. Lancet Planet. Health.

[170] Li P., Oyang X., Xie X., Guo Y., Li Z., Xi J., Zhu D., Ma X., Liu B., Li J. (2020). Perfluorooctanoic acid and perfluorooctane sulfonate co-exposure induced changes of metabolites and defense pathways in lettuce leaves. Environ. Pollut..

[171] Karbassiyazdi E., Fattahi F., Yousefi N., Tahmassebi A., Taromi A.A., Manzari J.Z., Gandomi A.H., Altaee A., Razmjou A. (2022). XGBoost model as an efficient machine learning approach for PFAS removal: Effects of material characteristics and operation conditions. Environ. Res..

[172] Jin H., Kong F., Li X., Shen J. (2024). Artificial intelligence in microplastic detection and pollution control. Environ. Res..

[173] Tang Y., Hardy T.J., Yoon J.Y. (2023). Receptor-based detection of microplastics and nanoplastics: Current and future. Biosens. Bioelectron..

[174] Zhang Y., Li J., Jiao S., Li Y., Zhou Y., Zhang X., Maryam B., Liu X. (2024). Microfluidic sensors for the detection of emerging contaminants in water: A review. Sci. Total Environ..

[175] Xu N., Lin H., Lin J.M., Cheng J., Wang P., Lin L. (2023). Microfluidic Chip-Based Modeling of Three-Dimensional Intestine-Vessel-Liver Interactions in Fluorotelomer Alcohol Biotransformation. Anal. Chem..

[176] Huang Y.N., Hsu C.N., Hou C.Y., Chen S.Y., Tain Y.L. (2024). Resveratrol Butyrate Esters Reduce Hypertension in a Juvenile Rat Model of Chronic Kidney Disease Exacerbated by Microplastics. Nutrients.

[177] Chen J., Spoljaric S., Calatayud-Sanchez A., Alvarez-Braña Y., Caruso F. (2023). Engineering Metal-Phenolic Network Nanoparticles via Microfluidics. ACS Appl. Mater. Interfaces.

[178] Gu Y., Jin L., Wang L., Ma X., Tian M., Sohail A., Wang J., Wang D. (2024). Preparation of Baicalin Liposomes Using Microfluidic Technology and Evaluation of Their Antitumor Activity by a Zebrafish Model. ACS Omega.

[179] Faramarzi P., Jang W., Oh D., Kim B., Kim J.H., You J.B. (2024). Microfluidic Detection and Analysis of Microplastics Using Surface Nanodroplets. ACS Sens..

[180] Akiyama Y., Egawa T., Koyano K., Moriwaki H. (2020). Acoustic Focusing of Microplastics in Microchannels: A Promising Continuous Collection Approach. Sens. Actuators B Chem..

[181] Liu S., Li Y., Shang L., Yin J., Qian Z., Chen C., Yang Y. (2022). Size-dependent neurotoxicity of micro- and nanoplastics in flowing condition based on an in vitro microfluidic study. Chemosphere.

[182] Liu X., Zhao Y., Dou J., Hou Q., Cheng J., Jiang X. (2022). Bioeffects of Inhaled Nanoplastics on Neurons and Alteration of Animal Behaviors through Deposition in the Brain. Nano Lett..

[183] Youssef K., Archonta D., Kubiseski T.J., Tandon A., Rezai P. (2021). Microfluidic Electric Parallel Egg-Laying Assay and Application to in-Vivo Toxicity Screening of Microplastics Using C. Elegans. Sci. Total Environ..

[184] Chen L., Zheng Y., Liu Y., Tian P., Yu L., Bai L., Zhou F., Yang Y., Cheng Y., Wang F. (2022). Microfluidic-Based in Vitro Thrombosis Model for Studying Microplastics Toxicity. Lab A Chip.

[185] Niu Y., Bai J., Kamm R.D., Wang Y., Wang C. (2014). Validating antimetastatic effects of natural products in an engineered microfluidic platform mimicking tumor microenvironment. Mol. Pharm..

[186] Kpeli G.W., Conrad K.M., Bralower W., Byrne C.E., Boue S.M., Burow M.E., Mondrinos M.J. (2024). Xenohormetic Phytochemicals Inhibit Neovascularization in Microphysiological Models of Vasculogenesis and Tumor Angiogenesis. Adv. Biol..

[187] Lee H., Kim D.S., Ha S.K., Choi I., Lee J.M., Sung J.H. (2017). A pumpless multi-organ-on-a-chip (MOC) combined with a pharmacokinetic-pharmacodynamic (PK-PD) model. Biotechnol. Bioeng..

[188] Chakraborty T.K., Rahman M.S., Nice M.S., Netema B.N., Islam K.R., Debnath P.C., Chowdhury P., Halder M., Zaman S., Ghosh G.C. (2024). Application of machine learning and multivariate approaches for assessing microplastic pollution and its associated risks in the urban outdoor environment of Bangladesh. J. Hazard. Mater..

[189] Xie H., Wei C., Wang W., Chen R., Cui L., Wang L., Chen D., Yu Y.L., Li B., Li Y.F. (2024). Screening the phytotoxicity of micro/nanoplastics through non-targeted metallomics with synchrotron radiation X-ray fluorescence and deep learning: Taking micro/nano polyethylene terephthalate as an example. J. Hazard. Mater..

[190] Ferreira R.O.G., Nag R., Gowen A., Xu J.L. (2024). Deciphering the cytotoxicity of micro- and nanoplastics in Caco-2 cells through meta-analysis and machine learning. Environ. Pollut..

[191] Liu C., Zong C., Chen S., Chu J., Yang Y., Pan Y., Yuan B., Zhang H. (2024). Machine learning-driven QSAR models for predicting the cytotoxicity of five common microplastics. Toxicology.

[192] Nayarisseri A., Khandelwal R., Tanwar P., Madhavi M., Sharma D., Thakur G., Speck-Planche A., Singh S.K. (2021). Artificial Intelligence, Big Data and Machine Learning Approaches in Precision Medicine & Drug Discovery. Curr. Drug Targets.

[193] Zothantluanga J.H., Chetia D., Rajkhowa S., Umar A.K. (2023). Unsupervised machine learning, QSAR modelling and web tool development for streamlining the lead identification process of antimalarial flavonoids. SAR QSAR Environ. Res..

[194] Qi X., Wang S., Fang C., Jia J., Lin L., Yuan T. (2025). Machine learning and SHAP value interpretation for predicting comorbidity of cardiovascular disease and cancer with dietary antioxidants. Redox Biol..

[195] Chen Y., Wang B., Zhao Y., Shao X., Wang M., Ma F., Yang L., Nie M., Jin P., Yao K. (2024). Metabolomic machine learning predictor for diagnosis and prognosis of gastric cancer. Nat. Commun..

[196] Chen L., Wang Y., Cai C., Ding Y., Kim R.S., Lipchik C., Gavin P.G., Yothers G., Allegra C.J., Petrelli N.J. (2024). Machine Learning Predicts Oxaliplatin Benefit in Early Colon Cancer. J. Clin. Oncol..

[197] Zhou Q., Wu F., Zhang W., Guo Y., Jiang X., Yan X., Ke Y. (2024). Machine learning-based identification of a cell death-related signature associated with prognosis and immune infiltration in glioma. J. Cell. Mol. Med..

[198] Guo F., Yang X., Hu C., Li W., Han W. (2023). Network Pharmacology Combined with Machine Learning to Reveal the Action Mechanism of Licochalcone Intervention in Liver Cancer. Int. J. Mol. Sci..

[199] Lu Y., Shan L., Cheng X., Zhu X.L. (2024). Exploring the mechanism underlying the therapeutic effects of butein in colorectal cancer using network pharmacology and single-cell RNA sequencing data. J. Gene Med..

[200] Liu Z., Luo C., Chen X., Feng Y., Feng J., Zhang R., Ouyang F., Li X., Tan Z., Deng L. (2024). Non invasive prediction of perineural invasion in intrahepatic cholangiocarcinoma by clinicoradiological features and computed tomography radiomics based on interpretable machine learning: A multicenter cohort study. Int. J. Surg..

[201] Lord C., Elsabbagh M., Baird G., Veenstra-Vanderweele J. (2018). Autism spectrum disorder. Lancet.

[202] Mohamed Nor N.H., Kooi M., Diepens N.J., Koelmans A.A. (2021). Lifetime Accumulation of Microplastic in Children and Adults. Environ. Sci. Technol..

[203] Skogheim T.S., Weyde K.V.F., Aase H., Engel S.M., Surén P., Øie M.G., Biele G., Reichborn-Kjennerud T., Brantsæter A.L., Haug L.S. (2021). Prenatal exposure to per- and polyfluoroalkyl substances (PFAS) and associations with attention-deficit/hyperactivity disorder and autism spectrum disorder in children. Environ. Res..

[204] Liu Z., Sokratian A., Duda A.M., Xu E., Stanhope C., Fu A., Strader S., Li H., Yuan Y., Bobay B.G. (2023). Anionic nanoplastic contaminants promote Parkinson’s disease-associated α-synuclein aggregation. Sci. Adv..

[205] Marin M., Annunziato K.M., Tompach M.C., Liang W., Zahn S.M., Li S., Doherty J., Lee J., Clark J.M., Park Y. (2024). Maternal PFOS exposure affects offspring development in Nrf2-dependent and independent ways in zebrafish (*Danio rerio*). Aquat. Toxicol..

[206] Sant K.E., Sinno P.P., Jacobs H.M., Timme-Laragy A.R. (2018). Nrf2a modulates the embryonic antioxidant response to perfluorooctanesulfonic acid (PFOS) in the zebrafish, *Danio rerio*. Aquat. Toxicol..

[207] Currie S.D., Benson D.B., Xie Z.R., Wang J.S., Tang L. (2025). Utilization of Artificial Intelligence Coupled with a High-Throughput, High-Content Platform in the Exploration of Neurodevelopmental Toxicity of Individual and Combined PFAS. J. Xenobiotics.

[208] Huang H., Hou J., Li M., Wei F., Liao Y., Xi B. (2025). Microplastics in the bloodstream can induce cerebral thrombosis by causing cell obstruction and lead to neurobehavioral abnormalities. Sci. Adv..

[209] Maenner M.J., Shaw K.A., Baio J., Washington A., Patrick M., DiRienzo M., Christensen D.L., Wiggins L.D., Pettygrove S., Andrews J.G. (2020). Prevalence of Autism Spectrum Disorder Among Children Aged 8 Years—Autism and Developmental Disabilities Monitoring Network, 11 Sites, United States, 2016. Morb. Mortal. Wkly. Rep. Surveill. Summ..

[210] Qian Q., Pu Q., Li L., Wu J., Cheng G., Cheng Y., Wang X., Wang H. (2025). Polylactic acid microplastics before and after aging induced neurotoxicity in zebrafish by disrupting the microbiota-gut-brain axis. J. Hazard. Mater..

[211] Chwabl P., Koppel S., Konigshofer P., Bucsics T., Trauner M., Reiberger T., Liebmann B. (2019). Detection of Various Microplastics in Human Stool: A Prospective Case Series. Ann. Intern. Med..

[212] Kumar P., Kumar A., Kumar D., Prajapati K.B., Mahajan A.K., Pant D., Yadav A., Giri A., Manda S., Bhandari S. (2025). Microplastics influencing aquatic environment and human health: A review of source, determination, distribution, removal, degradation, management strategy and future perspective. J. Environ. Manag..

[213] Desalegn A.A., van der Ent W., Lenters V., Iszatt N., Stigum H., Lyche J.L., Berg V., Kirstein-Smardzewska K.J., Esguerra C.V., Eggesbø M. (2023). Perinatal exposure to potential endocrine disrupting chemicals and autism spectrum disorder: From Norwegian birth cohort to zebrafish studies. Environ. Int..

[214] Liu J., Zhang X., Ren Q., Song C., Yu J., Cai Y., Chen D. (2024). Negative response to immunotherapy in dMMR or MSI-H gastric cancer with APC and PTEN mutations: A case report. Front. Oncol..

[215] Shitara K., Muro K., Watanabe J., Yamazaki K., Ohori H., Shiozawa M., Takashima A., Yokota M., Makiyama A., Akazawa N. (2024). Baseline ctDNA gene alterations as a biomarker of survival after panitumumab and chemotherapy in metastatic colorectal cancer. Nat. Med..

[216] Jang H., Chen J., Iakoucheva L.M., Nussinov R. (2023). Cancer and Autism: How PTEN Mutations Degrade Function at the Membrane and Isoform Expression in the Human Brain. J. Mol. Biol..

[217] Cummings K., Watkins A., Jones C., Dias R., Welham A. (2022). Behavioural and psychological features of PTEN mutations: A systematic review of the literature and meta-analysis of the prevalence of autism spectrum disorder characteristics. J. Neurodev. Disord..

[218] Busch R.M., Srivastava S., Hogue O., Frazier T.W., Klaas P., Hardan A., Martinez-Agosto J.A., Sahin M., Eng C., on behalf of the Developmental Synaptopathies Consortium (2019). Neurobehavioral phenotype of autism spectrum disorder associated with germline heterozygous mutations in PTEN. Transl. Psychiatry.

[219] Smith I.N., Dawson J.E., Eng C. (2023). Comparative Protein Structural Network Analysis Reveals C-Terminal Tail Phosphorylation Structural Communication Fingerprint in PTEN-Associated Mutations in Autism and Cancer. J. Phys. Chem. B.

[220] Wang S., Wu H., Shi X., Wang Y., Xu S. (2023). Polystyrene microplastics with different sizes induce the apoptosis and necroptosis in liver through the PTEN/PI3K/AKT/autophagy axis. Sci. Total Environ..

[221] Li Y., Ye Y., Zhu X., Li S., Rihan N., Yao Z., Sun Z., Gao P., Zhao Y., Lai Q. (2024). Polystyrene nanoplastics induce apoptosis, histopathological damage, and glutathione metabolism disorder in the intestine of juvenile East Asian river prawns (*Macrobrachium nipponense*). Sci. Total Environ..

[222] Li F., Gong X., Zhou Y., Geng Q., Jiang Y., Yao L., Qu M., Tan Z. (2024). Integrated evidence of transcriptional, metabolic, and intestinal microbiota changes in *Ruditapes philippinarum* due to perfluorooctanoic acid-induced immunotoxicity. Sci. Total Environ..

[223] Hong J., Du K., Jin H., Chen Y., Jiang Y., Zhang W., Chen D., Zheng S., Cao L. (2024). Evidence of promoting effects of 6:2 Cl-PFESA on hepatocellular carcinoma proliferation in humans: An ideal alternative for PFOS in terms of environmental health?. Environ. Int..

[224] Zhang M., Yu J., Liu A., Liu Q.Q., Sun T., Li X., Du Y., Li J., Wang B., Yang Q. (2023). Luteolin in the Qi Bi Anshen decoction improves propionic acid-induced autism-like behavior in rats by inhibiting LRP1/MMP9. Phytomedicine.

[225] Alsubaiei S.R.M., Alfawaz H.A., Bhat R.S., El-Ansary A. (2023). Nutritional Intervention as a Complementary Neuroprotective Approach against Propionic Acid-Induced Neurotoxicity and Associated Biochemical Autistic Features in Rat Pups. Metabolites.

[226] Gai J., Xing J., Wang Y., Lei J., Zhang C., Zhang J., Tang J. (2022). Exploration of potential targets and mechanisms of Naringenin in treating autism spectrum disorder via network pharmacology and molecular docking. Medicine.

[227] Xing X., Zhang J., Wu K., Cao B., Li X., Jiang F., Hu Z., Xia K., Li J.-D. (2019). Suppression of Akt-mTOR pathway rescued the social behavior in *Cntnap2*-deficient mice. Sci. Rep..

[228] Viana C.E., Bortolotto V.C., Araujo S.M., Dahleh M.M.M., Machado F.R., de Souza Pereira A., Moreira de Oliveira B.P., Leimann F.V., Gonçalves O.H., Prigol M. (2023). Lutein-loaded nanoparticles reverse oxidative stress, apoptosis, and autism spectrum disorder-like behaviors induced by prenatal valproic acid exposure in female rats. Neurotoxicology.

[229] Kisku A., Nishad A., Agrawal S., Paliwal R., Datusalia A.K., Gupta G., Singh S.K., Dua K., Sulakhiya K. (2024). Recent developments in intranasal drug delivery of nanomedicines for the treatment of neuropsychiatric disorders. Front. Med..

[230] Lu S., Zhu X., Zeng P., Hu L., Huang Y., Guo X., Chen Q., Wang Y., Lai L., Xue A. (2024). Exposure to PFOA, PFOS, and PFHxS induces Alzheimer’s disease-like neuropathology in cerebral organoids. Environ. Pollut..

[231] Cheng W., Li X., Zhou Y., Yu H., Xie Y., Guo H., Wang H., Li Y., Feng Y., Wang Y. (2022). Polystyrene microplastics induce hepatotoxicity and disrupt lipid metabolism in the liver organoids. Sci. Total Environ..

